# Ciclopirox Olamine Inhibits the NLRP3 Inflammasome to Alleviate Inflammatory Diseases

**DOI:** 10.1002/advs.75704

**Published:** 2026-05-19

**Authors:** Xinyu Xia, Hui You, Ke Zhang, Hui Jiang, Hongping Liu, Lianghua Liu, Aijie Zhang, You Zhou

**Affiliations:** ^1^ Department of Medical Laboratory Suining Central Hospital Suining China; ^2^ Department of Ophthalmology Suining Central Hospital Suining China; ^3^ The biology bank of Suining Central Hospital Suining Central Hospital Suining China; ^4^ Basic Laboratory Suining Central Hospital Suining China

**Keywords:** Ciclopirox olamine, inflammatory diseases, NLRP3 inflammasome

## Abstract

The aberrant activation of the NOD‐like receptor protein 3 (NLRP3) inflammasome has been implicated in the pathogenesis of various human inflammatory diseases. Although a wide variety of NLRP3 inflammasome inhibitors have been developed, no drug targeting the NLRP3 inflammasome has been approved for use in clinical settings. In this study, we identified ciclopirox olamine (CPX), an antifungal agent approved by the US Food and Drug Administration (FDA), as a novel NLRP3 inflammasome inhibitor. CPX specifically blocks NLRP3 inflammasome activation but not AIM2, NLRC4, Pyrin, NLRP1, or NLRP6 inflammasomes. Mechanistically, CPX directly disturbs NLRP3 inflammasome assembly by inhibiting NLRP3 oligomerization. Furthermore, CPX binds to the NACHT domain of NLRP3 at Y381 and reduces NLRP3 ATPase activity, thereby blocking NLRP3 oligomerization. More importantly, CPX administration notably exerts therapeutic effects on mouse models of sepsis, colitis, and metabolic disorders. CPX is also active ex vivo in cells from healthy individuals or patients with gout. Taken together, our results demonstrate that CPX acts as an NLRP3 inflammasome inhibitor and is a promising therapeutic agent for NLRP3 inflammasome‐associated diseases.

## Graphical abstract

There is no drug targeting the NLRP3 inflammasome that has been approved for use in clinical settings. Ciclopirox olamine (CPX), an antifungal agent approved by the US Food and Drug Administration (FDA), is identified as a specific and potent NLRP3 inflammasome inhibitor. CPX targets the NACHT domain of NLRP3 at Y381 to impair NLRP3 oligomerization and subsequent NLRP3 inflammasome assembly and full activation. CPX administration notably exerts therapeutic effects on mouse models of sepsis, colitis, and metabolic disorders. So, CPX is a promising therapeutic agent for NLRP3 inflammasome‐associated diseases.

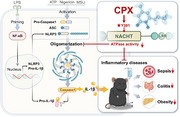

## 1 Introduction

The NOD‐like receptor protein 3 (NLRP3) inflammasome is a multi‐protein cytosolic immune sensor, comprising NLRP3, apoptosis‐associated speck‐like protein containing a CARD (ASC), and cysteinylaspartate‐specific proteinase 1 (caspase‐1) [1, 2, 3]. Upon stimulation, these components oligomerize and form a complex to promote NLRP3 inflammasome activation, which results in caspase‐1 activation and subsequent maturation of IL‐1β and IL‐18, and induces gasdermin‐D (GSDMD)‐dependent cell death [4, 5]. Unlike other immune sensor proteins, the NLRP3 inflammasome can sense a wide range of biological factors derived not only from the pathogen but also from the host or environment [6, 7, 8]. Consequently, the aberrant activation of the NLRP3 inflammasome is involved in a variety of human diseases, such as atherosclerosis [9], gout [10], type 2 diabetes [11], and Alzheimer's disease [12]. A growing number of studies have demonstrated that inhibiting the NLRP3 inflammasome is a promising therapeutic approach for these diseases [13, 14, 15].

To explore strategies for inhibiting the NLRP3 inflammasome, researchers have developed various small‐molecule inhibitors that target NLRP3 inflammasome components, including NLRP3, ASC, and caspase‐1, such as oridonin [16], CY‐09 [17], MCC950 [18], spirodalesol analog 8A [19], and sennoside A [20]. Moreover, NEK7, an essential promoter of NLRP3 inflammasome assembly, has been used as a target for developing drugs to inhibit NLRP3 inflammasome activation, such as entrectinib [21] and licochalcone B [22] In addition to these components, GSDMD, a mediator for downstream effects of NLRP3 inflammasome activation, is a target for drugs such as NU6300 [23] and dimethyl fumarate [24], m which treat NLRP3 inflammasome‐driven diseases. However, to date, some of these inhibitors have failed in clinical trials, while others are undergoing lengthy preclinical explorations. Therefore, no NLRP3 inflammasome inhibitor has received clinical approval as yet. Therefore, the search for efficient, specific, and safe NLRP3 inflammasome inhibitors remains a pressing need.

Repurposing well‐researched preclinical, clinical, and approved drugs has the greatest potential to swiftly transform a drug candidate from a laboratory study to a clinical application. The function requalification of US Food and Drug Administration (FDA)‐approved drugs provides a promising approach to rapidly develop potential inflammatory disease inhibitors with high efficiency and safety. Ciclopirox olamine (CPX) is a hydroxypyridone derivative that has been confirmed as an efficient fungal inhibitor and approved by the FDA as an antifungal agent for decades [25]. CPX has also shown other beneficial effects, including strong anticancer effects by inducing cellular reprogramming [26] or cancer cell apoptosis [27]. Moreover, clinical studies have shown that CPX displays biological activity in patients with advanced hematologic malignancies [28]. CPX improves polycystic kidney disease by inducing ferritinophagy and reducing cyst burden [29]. A recent study reported that CPX relieves inflammatory responses in ischemic stroke [30]. CPX possesses a high safety profile and is well tolerated by patients, with no observable cumulative toxicity, even at 40 mg/m^2^ orally once daily [28]. However, it remains unclear whether CPX inhibits NLRP3 inflammasome activation and mitigates NLRP3 inflammasome‐driven diseases.

In this study, we identified CPX as a potent and specific antagonist of the NLRP3 inflammasome by screening a library of 190 FDA‐approved drugs. Mechanistically, CPX directly targets the NACHT domain of NLRP3 at Y381, thereby blocking NLRP3 oligomerization and subsequent inflammasome assembly and full activation. Notably, CPX has demonstrated protective effects against sepsis, colitis, and metabolic disorders in mouse models, suggesting that CPX is a potential therapeutic agent for NLRP3 inflammasome‐driven diseases.

## 2 Results

### 2.1 Identification of CPX as a Potential NLRP3 Inflammasome Inhibitor

**FIGURE 1:**
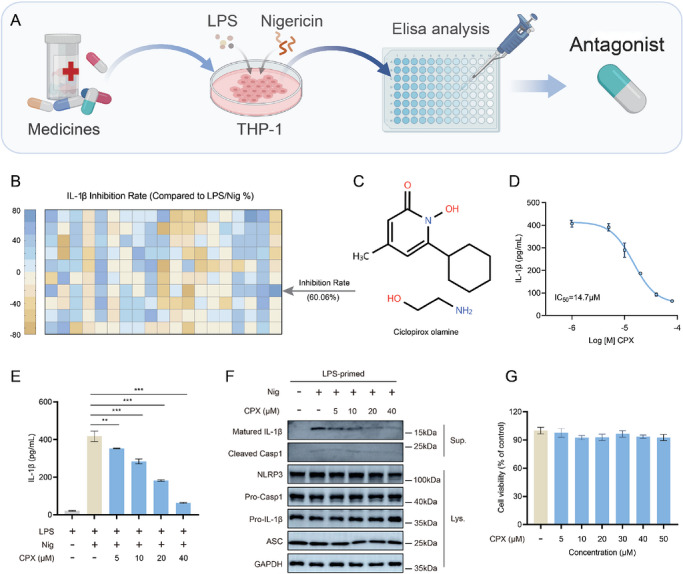
Identification of CPX as a potential inhibitor of NLRP3 Inflammasome. (A) Screening workflow of 190 FDA‐approved drugs. The THP‐1 cells (pretreated with 200 ng/mL PMA overnight) were primed with LPS, and incubated with drugs (10 µm) for 1 h prior to nigericin stimulation. Then, IL‐1β secretion was quantified by ELISA. (B) IL‐1β inhibition rate in culture supernatants (Sup) from THP‐1 cells. (C) The chemical structure of CPX. (D) IC_50_ of CPX on the NLRP3 inflammasome‐dependent IL‐1β secretion (0–80 µm). (E,F) LPS‐primed THP‐1 cells were treated with various doses of CPX or DMSO for 1 h and then stimulated with nigericin. (E) ELISA of IL‐1β in Sup from THP‐1 cells. (F) Immunoblot analysis of matured‐IL‐1β and cleaved caspase‐1 in Sup, and NLRP3, pro‐IL‐1β, pro‐caspase‐1, and ASC in lysates (Lys) from THP‐1 cells. (G) The cytotoxicity of CPX on THP‐1 cells was measured using the CCK‐8 kit. Data from three independent experiments (E. Values are mean ± SD) or are representative of three independent experiments(F). Statistical analyses were carried out via one‐way ANOVA for (E), ^**^ *p* < 0.01, ^***^ *p* < 0.001.

To identify new effective agents for the NLRP3 inflammasome, we screened a library approved by the FDA including 190 drugs in our laboratory via measuring IL‐1β secretion induced by the NLRP3 inflammasome agonist nigericin (Figure 1A,B; Figure S1), just as described assay (assay S1). By evaluating the inhibition efficiency, CPX was found to dramatically depress IL‐1β secretion (Figure 1B). Subsequently, we tested the half‐maximal inhibitory concentration (IC50) of CPX for IL‐1β secretion and found that its IC50 was approximately 14.70 µm (Figure 1D). CPX suppressed caspase‐1 cleavage and IL‐1β secretion in response to nigericin in a dose‐dependent manner (Figure 1E,F). Cell viability assays indicated that CPX did not exhibit any cytotoxicity at doses below 50 µm in THP‐1 cells (Figure 1G). Similarly, CPX was nontoxic in in vivo experiments under a universal dosage (Figure S2). Taken together, these results indicate that CPX is a potential NLRP3 inflammasome inhibitor.

### 2.2 CPX Blocks NLRP3 Inflammasome Activation

**FIGURE 2:**
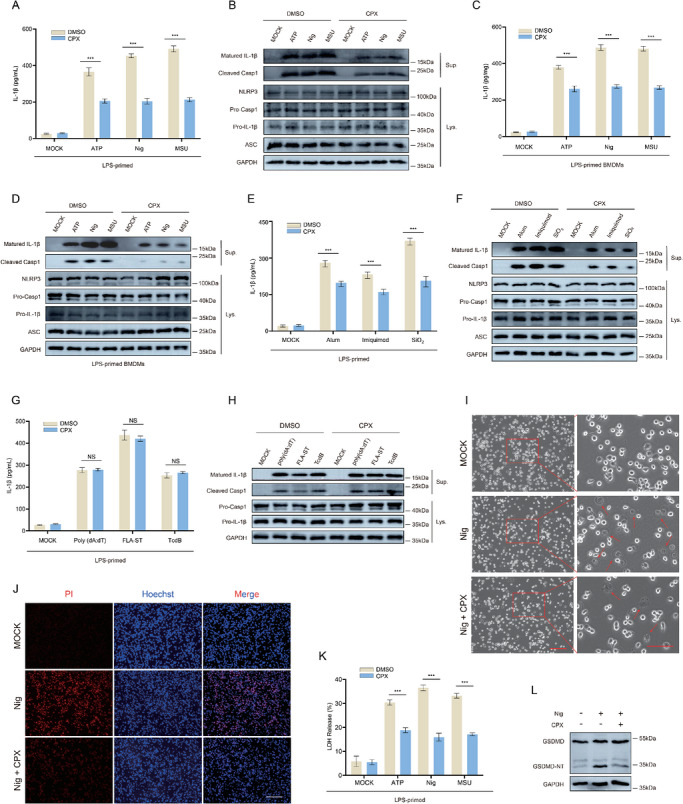
CPX blocks NLRP3 inflammasome activation. (A,B) THP‐1 cells were first primed with LPS (3 h) and then treated with or without 20 µm CPX for 1 h, followed by stimulation with ATP for 40 min, nigericin for 1 h, or MSU for 6 h. ELISA of IL‐1β in Sup (A), or immunoblot analysis of matured‐IL‐1β and cleaved caspase‐1 in Sup. (C,D) LPS‐primed BMDMs were treated with 20 µm CPX and then stimulated with ATP for 40 min, nigericin for 1 h, or MSU for 6 h. ELISA of IL‐1β in Sup (C), or immunoblot analysis of matured‐IL‐1β and cleaved caspase‐1 in Sup (D). (E‐F) THP‐1 cells were primed with LPS (3 h) and then treated with or without 20 µm CPX for 1 h, followed by stimulation with Alum for 6 h, imiquimod for 3 h, or SiO2 for 6 h. ELISA of IL‐1β in Sup (E), or immunoblot analysis of matured‐IL‐1β and cleaved caspase‐1 in Sup(F). (G,H) THP‐1 cells were treated and primed with LPS (3 h) and then treated with or without 20 µm CPX for 1 h, followed by stimulation with poly(dA:dT) for 4 h, FLA‐ST for 4 h, or TcdB for 1 h. ELISA of IL‐1β in Sup (G) or immunoblot analysis of matured‐IL‐1β and cleaved caspase‐1 in Sup (H). (I) Representative microscopic images of LPS‐primed THP‐1 cells upon nigericin stimulation with or without CPX pre‐treatment (red arrows mark pyroptotic cells). Scale bars correspond to 100 µm. (J) PI (red fluorescence) and Hoechst (blue fluorescence) staining of THP‐1 cells revealed the dead cells. Scale bars correspond to 100 µm. (K) The release of LDH from LPS‐primed THP‐1 cells, pre‐treated with or without CPX and then stimulated with ATP, nigericin, or MSU. (L) Immunoblot analysis of GSDMD cleavage from THP‐1 cells, primed with LPS before treatment with or without CPX, followed by treatment with nigericin. Data from three independent experiments (A,C,E,G,K. Values are mean ± SD) or are representative of three independent experiments with similar results (B,D,F,H–J). Statistical analyses were carried out via two‐way ANOVA for (A,C,E,G,K), ^***^ *p* < 0.001, NS not significant.

To explore the effect of CPX on NLRP3 inflammasome activation, we applied different NLRP3 inflammasome agonists to induce cell models. In lipopolysaccharide (LPS)‐primed human THP‐1 cells treated with nigericin, ATP, or monosodium urate crystals (MSU), CPX significantly reduced caspase‐1 cleavage and IL‐1β secretion (Figure 2A,B). The inhibitory effect of CPX, was measured in a mouse macrophage model. Primary mouse bone marrow‐derived macrophages (BMDMs) were primed with LPS and treated with nigericin, ATP, or MSU to activate the NLRP3 inflammasome. CPX administration reduced caspase‐1 cleavage and IL‐1β secretion in BMDMs (Figure 2C,D). To further validate the inhibitory effect of CPX, we tested its effect on NLRP3 inflammasome activation triggered by other agonists, including imiquimod, alum, and SiO_2_. CPX also blocked caspase‐1 cleavage and IL‐1β secretion induced by these agonists (Figure 2E,F). Considering that MCC950 is a well‐accepted NLRP3 inflammasome inhibitor, we compared the differences between CPX and MCC950 in inhibiting NLRP3 inflammasome‐driven IL‐1β secretion and cell death. Our data showed that the inhibitory effect of CPX at micromolar concentrations is similar to that of MCC950 at nanomolar concentrations in IL‐1β secretion and LDH release (Figure S3). Given that IL‐1β secretion could be triggered by multiple inflammasomes [31], we next sought to determine whether CPX specifically suppresses NLRP3 inflammasome activation. To this end, we transfected Poly(A/T) into cells to activate the AIM2 inflammasome, and employed FLA‐ST to activate the NLRC4 inflammasome and *Clostridium difficile toxin B* to activate the pyrin inflammasome [31, 32]. Our results showed that CPX had no visible inhibitory effects on these inflammasomes (Figure 2G,H). Meanwhile, we also found that CPX did not suppress NLRP1 inflammasome and NLRP6 inflammasome activation (Figure S4). These results suggest that CPX specifically inhibits NLRP3 inflammasome activation. In addition to caspase‐1 cleavage and IL‐1β secretion, NLRP3 inflammasome activation can contribute to GSDMD‐mediated pyroptosis [23, 24]. Some characteristic morphological changes related to pyroptosis were decreased following CPX administration (Figure 2I). Moreover, PI uptake and LDH release assays showed that CPX attenuated pyroptosis induced by NLRP3 inflammasome agonists (Figure 2J,K; Figure S5). Consistent with the above results, CPX treatment blocked the formation of GSDMD‐N (Figure 2L). Collectively, these results demonstrate that CPX is a specific and potent inhibitor of the NLRP3 inflammasome.

### 2.3 CPX Inhibits NLRP3 Inflammasome Activation In Vivo and in Patient Cells

**FIGURE 3:**
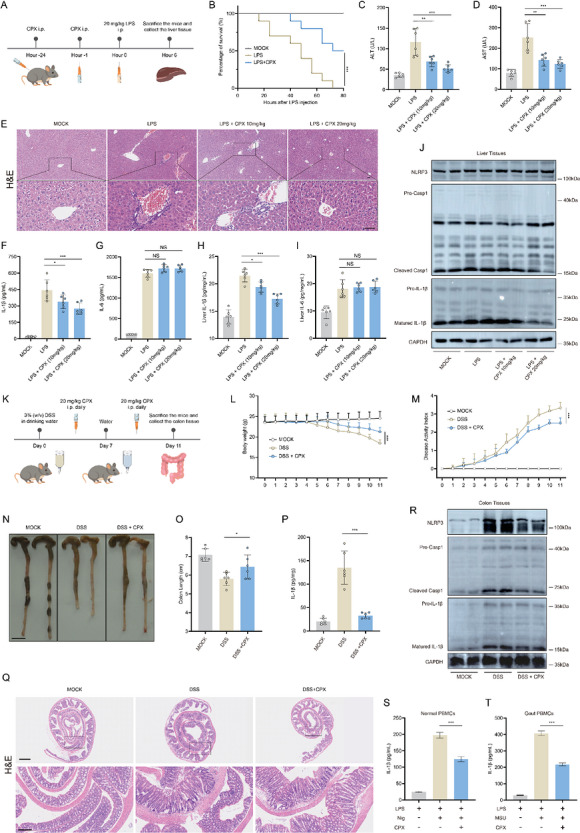
CPX inhibits NLRP3 inflammasome activation in vivo and in patient cells. (A–J) C57BL/6 mice were intraperitoneally injected with vehicle, CPX (10 mg/kg), or CPX (20 mg/kg) for 1 h prior to LPS injection to induce sepsis. (A) Schematic diagram of the LPS‐induced sepsis model. (B) Survival analysis of mice pretreated with vehicle or CPX (20 mg/kg) for 1 h before being intraperitoneally injected with LPS (*n* = 10). (C,D) The activity of plasma ALT and AST (*n* = 6). (E) Representative images of H&E‐stained liver sections. Scale bars, 50 µm. (F–I) ELISA of IL‐1β and IL‐6 in serum and in Sup from cultured liver tissues (*n* = 6). (F) IL‐1β and (G) IL‐6 levels in serum. (H) IL‐1β and (I) IL‐6 levels in liver tissues. (J) Immunoblot analysis of matured‐IL‐1β and cleaved caspase‐1 in liver tissues. (K–R) C57BL/6 mice were treated with 3% DSS in drinking water for 6 days, followed by 4 days of normal drinking water. CPX or vehicle was administered daily (*n* = 6). (K) Schematic diagram of DSS‐induced colitis model. (L) Body weights, (M) the disease activity index (DAI), (N) representative colon images, and (O) colon length were measured. (P) ELISA of IL‐1β in colon tissues. (Q) H&E‐stained colon tissues. (R) Immunoblot analysis of matured‐IL‐1β and cleaved caspase‐1 in colon tissues. (S,T) PBMCs primed with LPS and treated with 20 µm CPX for 1 h, and then stimulated with nigericin or MSU. ELISA of IL‐1β in Sup of PBMCs isolated from healthy individuals (S) or gout patients (T). Date from three independent experiments (S,T. Values are mean ± SD). Statistical analyses were carried out via two‐way ANOVA for (B–D, F–L, L,M, O,P, S,T), ^*^ *p* < 0.05, ^**^ *p* < 0.01, ^***^ *p* < 0.001, NS not significant.

Considering that CPX showed a potent inhibitory effect on the NLRP3 inflammasome in vitro, we subsequently investigated its function in vivo. Excessive NLRP3 inflammasome activation accelerates inflammatory responses in LPS‐induced sepsis, resulting in multiple organ failure and mortality [33]. Therefore, mice were treated with or without CPX before LPS challenge, and the results showed that CPX treatment significantly improved the survival of LPS‐induced mice (Figure 3B). We also measured liver damage markers and found that CPX reduced alanine aminotransferase (ALT) and serum aspartate aminotransferase (AST) levels and improved hepatic pathological injury (Figure 3C–E). CPX treatment inhibited IL‐1β but had no inhibitory effect on IL‐6 in the serum and liver (Figure 3F–I). Furthermore, CPX weakened caspase‐1 cleavage and IL‐1β secretion in liver tissue under LPS treatment (Figure 3J).

Aberrant activation of the NLRP3 inflammasome has been considered an encourager in a chemically induced colitis model [34]. Therefore, we next tested the therapeutic potential of CPX in dextran sulfate sodium (DSS)‐induced colitis. The results revealed that CPX treatment significantly reduced weight loss and improved the accumulated disease activity index induced by DSS (Figure 3L,M). Additionally, CPX prevented colon length shortening in DSS‐induced colitis (Figure 3N,O), a characteristic feature of colitis. Hematoxylin and eosin (H&E) staining of colonic histopathology showed that CPX improved colonic pathological damage by mitigating DSS‐induced crypt disappearance and inflammatory cell influx (Figure 3Q). Additionally, CPX administration markedly suppressed DSS‐induced caspase‐1 cleavage and IL‐1β secretion in the colon (Figure 3P,R). These results highlight the therapeutic effect of CPX in acute colitis.

To confirm the therapeutic effect of CPX on NLRP3 inflammasome‐related diseases, we measured its inhibitory effect in human cells using two approaches. First, we collected peripheral blood mononuclear cells (PBMCs) from healthy individuals and subsequently induced NLRP3 inflammasome activation by LPS plus nigericin. We found that CPX markedly suppressed nigericin‐induced IL‐1β secretion in PBMCs from healthy individuals (Figure 3S). Using the same approach, we found the same effects on IL‐1β secretion in PBMCs from patients with gout (Figure 3T). Taken together, the above results strongly indicate that CPX also suppresses the NLRP3 inflammasome in vivo and has therapeutic potential in inflammatory diseases.

### 2.4 CPX Inhibits NLRP3 Inflammasome Assembly

**FIGURE 4:**
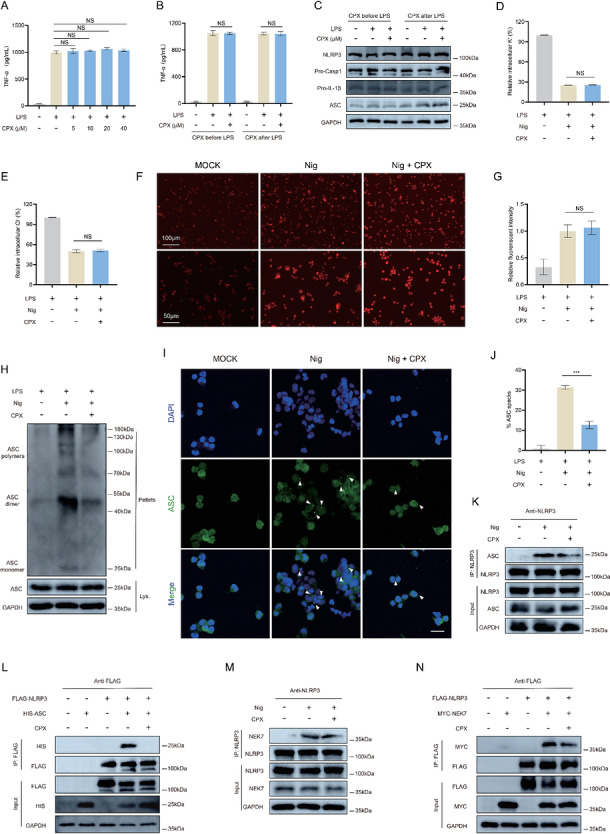
CPX inhibits NLRP3 inflammasome assembly. (A) ELISA of TNF‐α in Sup from THP‐1 cells treated with various doses of CPX and then stimulated with 500 ng/mL LPS for 3 h. (B) ELISA of TNF‐α and (C) immunoblot analysis of NLRP3, pro‐caspase‐1, pro‐IL‐1β, and ASC in Lys from THP‐1 cells treated with 20 µm CPX before or after stimulation with 500 ng/mL LPS for 3 h. (D,E) Qualification of potassium and chloride ions in LPS‐primed THP‐1 cells pretreated with or without 20 µm CPX and then stimulated with nigericin. (F,G) Qualification of mtROS in THP‐1 cells was primed with LPS, pre‐treated with or without CPX, and subsequently stimulated with nigericin. (H) Immunoblot analysis of ASC oligomerization. (I) Representative images of ASC specks detected by immunofluorescence. DAPI shows nuclei, and white arrows show an example of ASC specks formed. Scale bar corresponds to 50 µm. (J) Percentage of ASC speck‐positive cells. (K) IP and immunoblot analysis to evaluate the NLRP3‐ASC interaction in LPS‐primed THP‐1 cells pretreated with CPX and then stimulated with nigericin. (L) IP and immunoblot analysis of HEK‐293T cells transfected with FLAG‐NLRP3, HIS‐ASC plasmids as indicated and treated with CPX. (M) IP and immunoblot analysis to evaluate the NLRP3‐NEK7 interaction in LPS‐primed THP‐1 cells pretreated with CPX and then stimulated with nigericin. (N) IP and immunoblot analysis of HEK‐293T cells transfected with FLAG‐NLRP3, MYC‐NEK7 plasmids as indicated, and treated with the CPX Data from three independent experiments(A,B,D,E,G,J. Values are mean ± SD) or are representative of three independent experiments with similar results(F,H,I,K–N). Statistical analyses were carried out via two‐way ANOVA for (A,B,D,E,G,J), ^***^ *p* < 0.001, NS not significant.

Next, we explored the underlying mechanism by which CPX blocks NLRP3 inflammasome activation. NLRP3 inflammasome activation is a two‐step sequential process, involving a priming signal followed by an activating signal. The priming stage initiates expression of the NLRP3 inflammasome. Our results showed that CPX did not inhibit ASC, NLRP3, pro‐IL‐1β, or pro‐caspase‐1 expression (Figure 2B). Considering that this stage is regulated by the NF‐κB pathway, we examined NF‐κB activation [1, 2, 35] and found that CPX did not affect the LPS‐induced phosphorylation of p65 and degradation of IκB‐α (Figure S6). Additionally, CPX, at several concentrations, did not reduce TNF‐α production, a cytokine directly driven by the NF‐κB pathway (Figure 4A). Furthermore, CPX administration did not suppress the TNF‐α production and NLRP3 inflammasome expression before or after LPS priming (Figure 4B,C). These results demonstrate that CPX cannot intervene in the LPS‐induced priming process.

We next focused on the activating signal, a stage of NLRP3 inflammasome activation controlled by numerous upstream signaling events, including potassium efflux and chloride ion efflux [36, 37]. Therefore, we investigated whether CPX could disturb these events. Our results showed that CPX did not impair nigericin‐induced potassium and chloride ion efflux (Figure 4D,E), nor did it affect excessive mitochondrial reactive oxygen species (ROS) generation (Figure 4F,G), which is another important upstream signaling event for the activating stage [38]. Collectively, these findings suggest that CPX has a minimal effect on the upstream signaling event for the NLRP3 inflammasome activating stage. ASC oligomerization and speck formation are essential for subsequent caspase‐1 cleavage, which is considered to be a sign of NLRP3 inflammasome assembly completion [4, 6]. Our results showed that CPX treatment markedly attenuated nigericin‐induced ASC oligomerization and speck formation (Figure 4H–J), indicating that CPX might break NLRP3 inflammasome assembly. The interaction between ASC and NLRP3 is a crucial step in NLRP3 inflammasome assembly [16, 17, 23]. CPX inhibited the endogenous and exogenous ASC‐NLRP3 interaction in THP‐1 and 293T cells (Figure 4K,L). Numerous studies have confirmed that NEK7 can directly bind to NLRP3 and that the NEK7‐NLRP3 interaction is indispensable for NLRP3 inflammasome assembly and subsequent activation [16, 21, 22, 36]. Interestingly, CPX also inhibited the NEK7‐NLRP3 interaction (Figure 4M,N). Collectively, these results demonstrate that CPX blocks NLRP3 inflammasome activation by inhibiting NLRP3 inflammasome assembly.

### 2.5 CPX Directly Targets NLRP3 and Inhibits its Oligomerization

**FIGURE 5:**
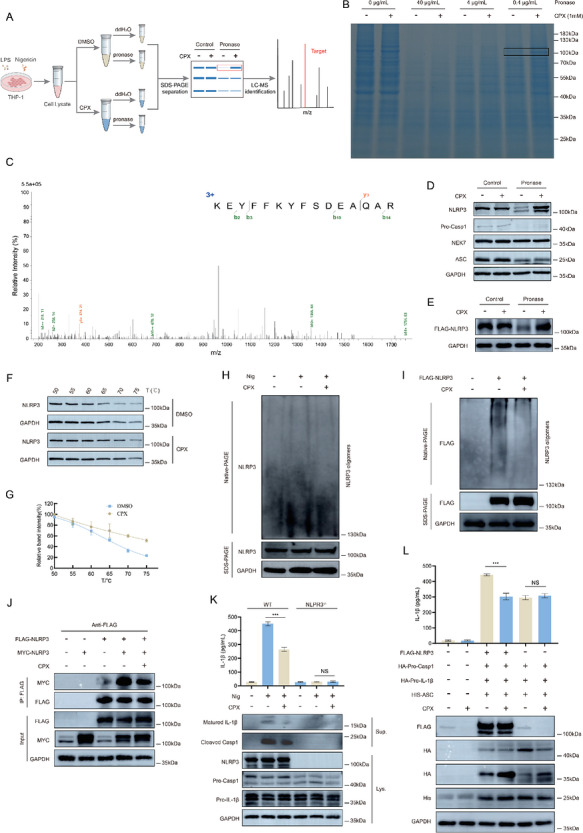
CPX directly targets NLRP3 and inhibits its oligomerization. (A) The schematic shows the steps for identifying the target of CPX in THP‐1 cells using drug affinity responsive target stability (DARTS) and mass spectrometry analysis. (B) Coomassie blue staining of DARTS assay using THP‐1 cell lysates treated with or without CPX. (C) The target protein of CPX was identified by mass spectrogram. (D) DARTS in THP‐1 cells incubation with CPX or DMSO. (E) DARTS in HEK‐293T cells transfected with the FLAG‐NLRP3 plasmid under incubation with CPX or DMSO. (F) CESTA of NLRP3 in THP‐1 cells incubated with CPX or DMSO. (G) The melting curve generated from CETSA. (H) The oligomerization of NLRP3 in THP‐1 cells treated with CPX was analyzed by native‐PAGE. (I) NLRP3 oligomerization in HEK‐293T cells expressing FLAG‐NLRP3 plasmids and treated with CPX. (J) IP and immunoblot analysis of HEK‐293T cells transfected with FLAG‐NLRP3 and MYC‐NLRP3 plasmids as indicated and treated with CPX. (K) ELISA of IL‐1β in Sup and immunoblot analysis of matured‐IL‐1β and cleaved caspase‐1 in Sup from *WT* and *NLRP3^−/−^* BMDMs were first primed with LPS and then treated with CPX, followed by stimulation with nigericin. (L) ELISA of IL‐1β in Sup and immunoblot analysis in Lys from HEK‐293T cells transfected with HA‐IL‐1β, HA‐pro‐caspase‐1, HIS‐ASC, with or without FLAG‐NLRP3 plasmids as indicated and treated with CPX. Data from three independent experiments (G,K,L. Values are mean ± SD) or are representative of three independent experiments with similar results (D,F,H–L). Statistical analyses were carried out via two‐way ANOVA for (K,L), ^**^ *p* < 0.01, ^***^ *p* < 0.001, NS not significant.

We then sought to explore the mechanism by which CPX mediated the disruption of NLRP3 inflammasome assembly. First, we analyzed the CPX target protein. Using the DARTS assay [39], we observed a dominant protective band at approximately 100–130 kD on the Coomassie brilliant blue staining gel in the CPX group (Figure 5A,B). Then, these differential bands from the gel were analyzed by mass spectrometry, and NLRP3 was detected as an interacting protein of CPX, as the iBAQ ratio is 7.15(Figure 5C; Figure S7), consistent with the dominant protective band. This result led us to hypothesize that NLRP3 may be the target of CPX. To address this speculation, we performed immunoblotting using DARTS samples to test other common target proteins for inhibiting NLRP3 inflammasome activation (pro‐caspase‐1, NEK‐7, and ASC), confirming that CPX only protected NLRP3 from protease hydrolysis (Figure 5D). We then conducted transfection experiments using Flag‐tagged NLRP3 in HEK‐293T cells and found that CPX also protected exogenous NLRP3 from protease hydrolysis (Figure 5E). Moreover, we performed a cellular thermal shift assay (CETSA), another established technique to investigate the interactions between drug and target protein within cellular contexts [40]. The results showed that CPX significantly increased the thermal stability of NLRP3 even at elevated temperatures compared with the DMSO‐treated group (Figure 5F,G), suggesting that CPX directly interacts with NLRP3.

Previous studies have reported that NLRP3 oligomerization is decisive for its interaction with ASC and NEK7, and inhibitors damage NLRP3 oligomerization by directly binding to NLRP3 [16, 41]. The inhibitory effect of CPX on endogenous and exogenous NLRP3 oligomerization was confirmed using Native‐PAGE or SDD‐PAGE, respectively, in THP‐1 cells and 293 T cells (Figure 5H,I; Figure S8). Furthermore, the direct NLRP3–NLRP3 interaction was measured by transfecting 293T cells with Flag‐NLRP3 and Myc‐NLRP3. The direct NLRP3–NLRP3 interaction was also hampered by CPX (Figure 5J).

To determine whether the inhibition of CPX on NLRP3 inflammasome activation was NLRP3‐dependent, we collected BMDMs from *NLRP3^−/−^* mice to conduct a cell model of NLRP3 inflammasome activation. The inhibitory effects of CPX on NLRP3 inflammasome activation were abolished in *NLRP3^−/−^* BMDMs (Figure 5K). Recent reports have shown that the expression of ASC, pro‐IL‐1β, and pro‐caspase‐1 also leads to IL‐1β secretion in 293T cells, and the addition of NLRP3 increases this secretion [42, 43]. Our results showed that CPX markedly reduced IL‐1β secretion in cells expressing NLRP3, but barely impacted IL‐1β secretion in cells lacking NLRP3 (Figure 5L). Based on these results, we concluded that CPX directly interacts with NLRP3 and inhibits its oligomerization, thereby blocking the assembly and full activation of the NLRP3 inflammasome.

### 2.6 CPX Binds to Tyrosine 381 of the NACHT Domain

**FIGURE 6:**
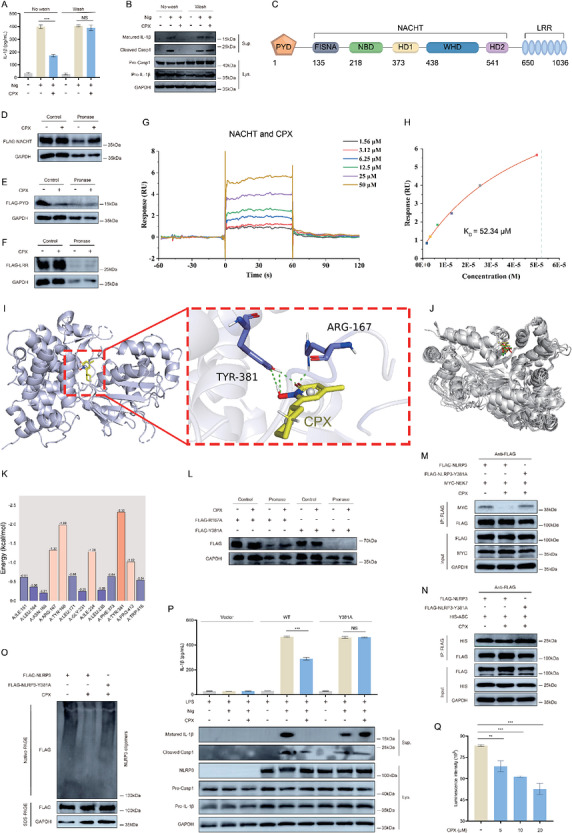
CPX binds to tyrosine 381 of the NACHT domain. (A,B) LPS‐primed THP‐1 cells were treated with CPX for 30 min and washed out, then stimulated with nigericin. (A) ELISA of IL‐1β in Sup. (B) Immunoblot analysis of matured‐IL‐1β and cleaved caspase‐1 in Sup. (C) Schematic of the protein domains of NLRP3. (D–F) DARTS in HEK293T transfected with (D) FLAG‐NACHT domain, (E) FLAG‐PYD domain, and (F) FLAG‐LRR domain plasmids under incubation with CPX or DMSO. (G,H) The binding affinity of CPX to the NLRP3‐NACHT domain was measured by SPR assay. (I) Molecular docking analysis of CPX bound to NLRP3‐NACHT domain. CPX (yellow, stick model) in the binding site of NLRP3‐NACHT domain (lavender, cartoon, and PDB ID: 7ALV). (J) Structural comparison of the complex at five moments (0, 25, 50, 75, 100 ns) during a 100 ns molecular dynamics simulation. (K) Energy contribution of amino acid residues involved in binding. (L) DARTS in HEK293T transfected with R167A NACHT domain or Y381A NACHT domain plasmids under incubation with CPX or DMSO. (M) IP and immunoblot analysis of HEK293T cells transfected with MYC‐NEK7, FLAG‐NLRP3, or Y381A mutant FLAG‐NLRP3 plasmids and treated with 20 µm CPX. (N) IP and immunoblot analysis of HEK293T cells transfected with HIS‐ASC, FLAG‐NLRP3, or Y381A mutant FLAG‐NLRP3 plasmids and treated with 20 µm CPX. (O) NLRP3 oligomerization in HEK‐293T cells transfected with FLAG‐NLRP3 or Y381A mutant FLAG‐NLRP3 plasmids and treated with CPX. (R) LPS‐primed NLRP3‐/‐ BMDMs reconstituted with WT or Y381A mutant NLRP3, were treated with CPX and then stimulated with nigericin. ELISA of IL‐1β in Sup, immunoblot analysis of matured‐IL‐1β and cleaved caspase‐1 in Sup. (Q) ATPase activity assay for purified human NLRP3 in the presence of different concentrations of CPX. Data from three independent experiments (A, P, Q. Values are mean ± SD) or are representative of three independent experiments with similar results (B, D–F, L–P). Statistical analyses were carried out via two‐way ANOVA for (A, P, Q), ^**^ *p* < 0.01, ^***^ *p* < 0.001, NS not significant.

To investigate the interaction between CPX and NLRP3, we sought to determine whether CPX's inhibition of NLRP3 inflammasome activation is reversible. After CPX treatment, cells were washed three times every 5 min to remove the agent and then stimulated with nigericin. We found that the inhibitory effect of CPX on NLRP3 inflammasome activation was dispelled in washed cells (Figure 6A,B), indicating that the inhibition of NLRP3 inflammasome activation by CPX is reversible. NLRP3 contains three functional domains (Figure 6C), including the carboxy‐terminal LRR, central NACHT domain, and amino‐terminal Pyrin domain (PYD) [17, 18]. Next, we tested which region was responsible for the binding between CPX and NLRP3 by expressing Flag‐NACHT, Flag‐PYD, and Flag‐LRR in 293T cells. Our results revealed that CPX protected only the NACHT domain of NLRP3 from protease hydrolysis (Figure 6D–F), suggesting that the NACHT domain was responsible for the interaction between NLRP3 and CPX. Moreover, we purified the protein of the NACHT domain and confirmed a high‐affinity interaction between CPX and the NACHT domain by SPR assay (Figure 6G,H).

To determine which residues on the NACHT domain bind to CPX, we first analyzed the interaction at the molecular level using docking software. Molecular docking data showed that CPX readily docked into the NLRP3 pocket, with a binding energy of −6.77 kcal/mol (Figure 6I,J), indicating that CPX can effectively bind to NLRP3. Moreover, CPX was predicted to bind to NLRP3 by hydrogen bonding at arginine 167 (R167) and tyrosine 381 (Y381) (Figure 6I). To further elucidate the detailed mode of the binding of CPX to the NACHT domain, the better‐docking pose of the NACHT domains was subjected to molecular dynamics simulation (MDS) for a time scale of 100 nanoseconds (ns). The Free Energy Landscape (FEL) serves to describe the energetically favored conformations throughout the dynamic simulation of the complex structure, and strong, and stable interactions result in the formation of nearly single and smooth energy clusters. The results also demonstrated that CPX and NLRP3 were compact and stable, and R167 and Y381 were the primary binding sites (Figure 6K; Figure S9A–G). The number of hydrogen bonds formed in the CPX‐NLRP3 complex is approximately one (Figure S9D), suggesting that the interaction may be reversible, which is consistent with the above results (Figure 6A). Next, using NACHT mutations in which an arginine or tyrosine was substituted with an alanine, we found that Y381A, but not R167A mutation, abolished the protective effect of CPX on NACHT from protease hydrolysis (Figure 6L), indicating that CPX may bind to Y381 of the NACHT domain. According to the above results, we also investigated whether Y381A NLRP3 abolished the suppressive effect of CPX on the process of NLRP3 inflammasome assembly. Our data showed that transfecting Y381A NLRP3 into 293T cells not only eliminated the suppressive effect of CPX on the interaction between NLRP3 and NEK7 or ASC but also abolished its inhibitory impact on NLRP3 oligomerization (Figure 6M–O). These results further suggest that CPX binds to Y381 of the NACHT domain.

Subsequently, we re‐expressed wild type (WT) NLRP3 or Y381A NLRP3 in BMDMs from *NLRP3^−/−^* mice, and found that CPX rarely affected NLRP3 inflammasome activation in Y381A mount cells (Figure 6P). These results indicate that CPX binds to Tyrosine 381 of NLRP3 to inhibit NLRP3 inflammasome activation.

The ATPase activity of the NACHT domain of NLRP3 is indispensable for NLRP3 oligomerization [17, 18]. A previous study reported that tyrosine 381 of NLRP3 is a binding site of ATP, and occupying the ATP‐binding site with inhibitors can inhibit ATPase activity [17, 44]. Thus, we tested whether CPX could affect NLRP3 ATPase activity. We found that CPX inhibited the ATPase activity of NLRP3 at doses of 5–20 µm (Figure 6Q). These results suggest that CPX inhibits NLRP3 ATPase by binding to tyrosine 381 and subsequently disturbs NLRP3 inflammasome assembly.

### 2.7 CPX Ameliorates HFD‐Induced Metabolic Disorders by Inhibition of NLRP3‐Dependent Inflammation

**FIGURE 7:**
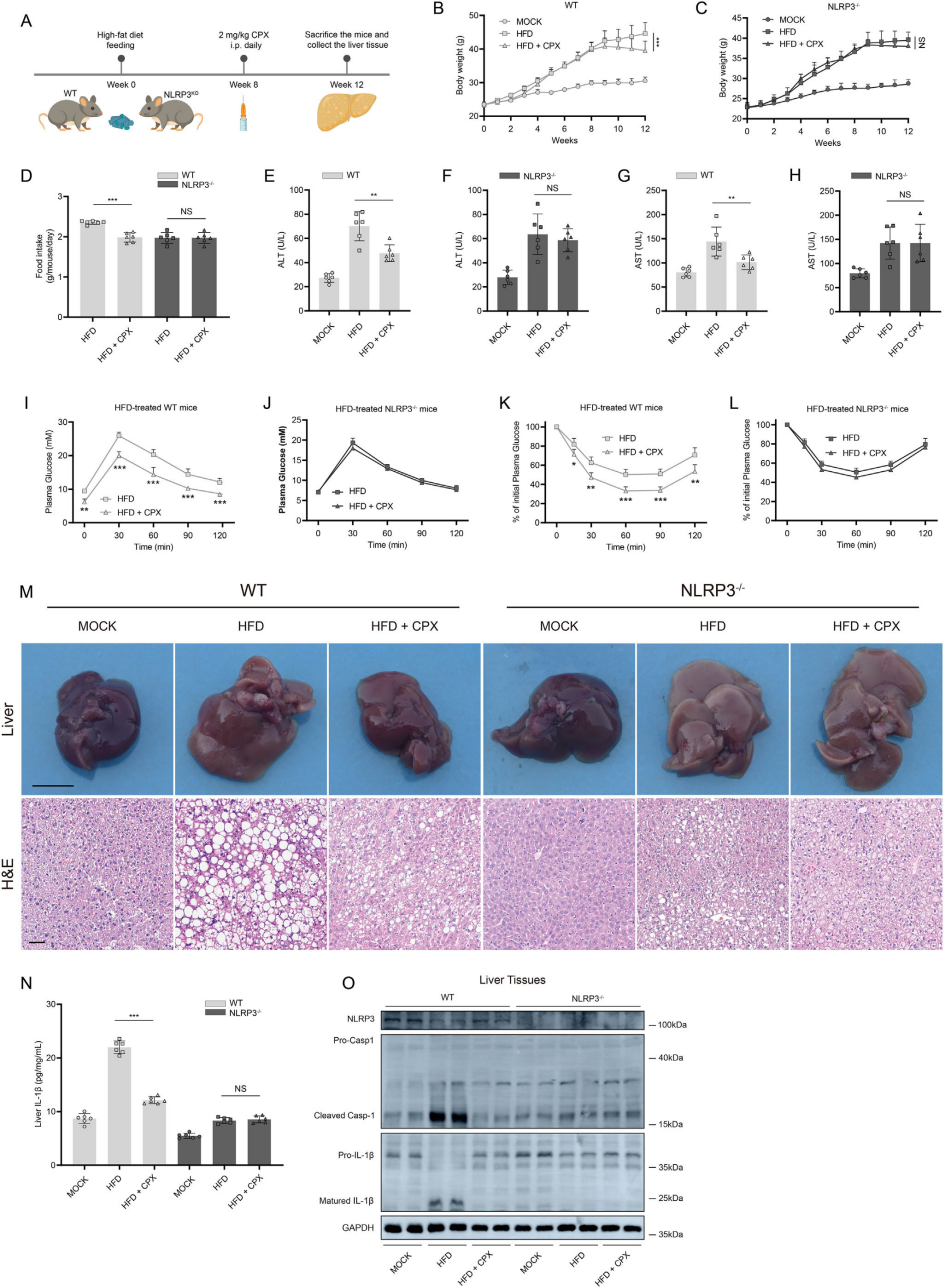
CPX ameliorates HDF‐induced metabolic disorders by inhibition of *NLRP*_3_‐dependent inflammation. (A–M) Wild‐type (WT) and *NLRP*_3_^−/−^ C57BL/6 were maintained on a HFD for 12 weeks throughout the experiment. Mice received a daily intraperitoneal injection of either vehicle or CPX (2 mg/kg) in the last 4 weeks (*n* = 6). (A) Experiment design of the HFD‐induced metabolic disorders model establishment and administration of CPX. Body wights of WT mice(B) and *NLRP*_3_^−/−^ mice(C) were measured at the indicated time points. (D) Daily food intake of WT and *NLRP*_3_^−/−^ mice. (E–H) The serum levels of ALT (E,F) and AST (G,H) of WT and *NLRP*_3_^−/−^ mice. (I–L) Glucose tolerance test (I,J) and insulin tolerance test (K,L) of WT and *NLRP*_3_^−/−^ mice. (M) Representative phenotype and H&E staining of livers of WT and *NLRP*_3_^−/−^ mice. Bar, 50 µm. (N) Liver tissues of WT and *NLRP*_3_^−/−^ mice were isolated and cultured for 24 h, and Sup were analyzed by ELISA for IL‐1β. (O) Immunoblot analysis of matured‐IL‐1β and cleaved caspase‐1 in liver tissues of WT and *NLRP*_3_^−/−^ mice. Statistical analyses were carried out via two‐way ANOVA for (B–L,N), ^**^ *p* < 0.01, ^***^ *p* < 0.001, NS not significant.

Recent studies have corroborated that NLRP3 inflammasome activation participates in high‐fat diet (HFD)‐induced metabolic disorders in mice [17, 21, 45]. Hence, we examined whether CPX administration could reverse HFD‐induced metabolic disorders in mice and whether this effect depended on NLRP3 inflammation inhibition. The WT mice and *NLRP3^−/−^* mice were fed an HFD for 12 weeks and then administered CPX once a day at a dose of 2 mg/kg for 4 weeks. CPX significantly reduced weight gain and food intake in HFD‐treated WT mice (Figure 7B–D; Figure S10). Moreover, CPX administration decreased ALT and AST levels in WT mice (Figure 7E,G). Additionally, the WT mice treated with CPX exhibited better insulin sensitivity than the controls (Figure 7K). However, these beneficial effects of CPX on HFD‐induced metabolic disorders were not observed in *NLRP3^−/−^* mice (Figure 7F,H,L). We also evaluated the effect of CPX on HFD‐induced hepatic steatosis and found that CPX administration significantly ameliorated HFD‐induced hepatic steatosis and intracellular lipid accumulation; however, this effect was barely observed in *NLRP3^−/−^* mice (Figure 7). These results indicated that the ability of CPX to relieve symptoms of HFD‐induced metabolic disorder symptoms in mice was NLRP3‐dependent. Then, we determined whether CPX could inhibit NLRP3 inflammasome activation in HFD‐induced metabolic disorders. As expected, NLRP3 inflammasome‐dependent caspase‐1 cleavage and IL‐1β secretion in liver tissue under HFD conditions were suppressed by CPX treatment (Figure 7N,O). Taken together, our results demonstrate that CPX reduces HFD‐induced metabolic disorders by inhibiting NLRP3 inflammasome activation.

**FIGURE 8:**
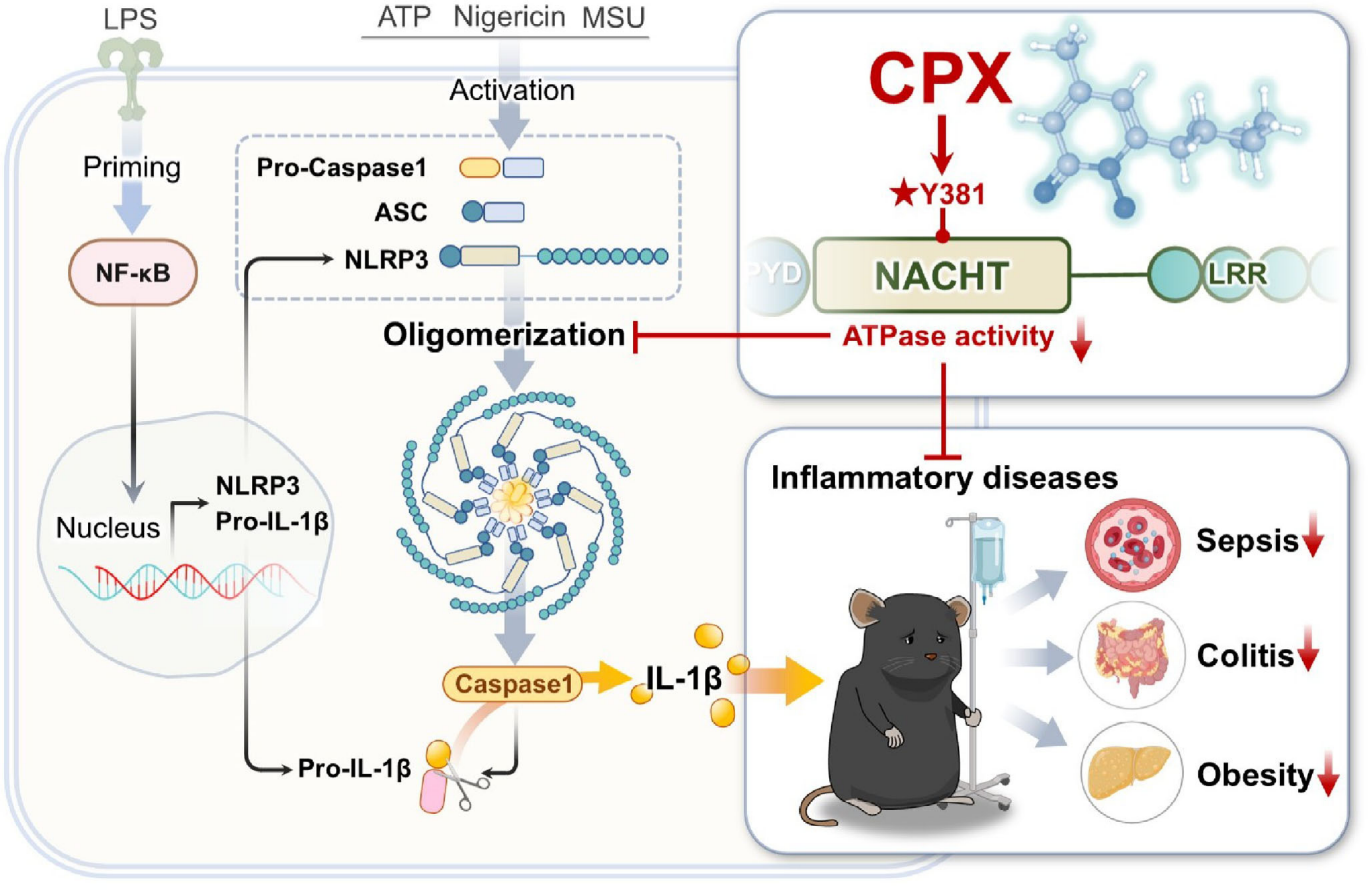
The mechanism of CPX blocking NLRP3 inflammasome activation.

Based on these experimental data, Figure 8 illustrates how CPX blocks NLRP3 inflammasome activation and contributes to the alleviation of related diseases.

## 3 Discussion

The NLRP3 inflammasome is an integral part of innate immunity that supports host defense; however, its aberrant activation seems to be closely related to multiple human inflammatory diseases. Increasing evidence strongly suggests that blocking activation of the NLRP3 inflammasome may serve as a potential and effective strategy for treating NLRP3 inflammasome‐related diseases [13, 14, 15, 46, 47]. Emerging strategies focus on developing inhibitors to eliminate NLRP3 inflammasome activation [16, 17, 18, 19, 20, 21, 22]. In this study, we identified CPX as a potent and specific inhibitor of the NLRP3 inflammasome through systematic screening of a library including 190 FDA‐approved drugs. In vivo experiments revealed that CPX also exhibited protective effects in both LPS‐induced sepsis and DSS‐induced colitis models. Mechanistic investigations indicated that CPX suppresses NLRP3 oligomer formation by targeting the NACHT domain of NLRP3. This inhibition disrupts the assembly of NLRP3 inflammasomes, thereby blocking NLRP3 inflammasome activation. Our findings suggest that CPX is a potent candidate drug for treating NLRP3‐associated inflammatory diseases.

Although an increasing number of small‐molecule compounds have been identified as NLRP3 inflammasome inhibitors, such as UK5099 [48], CY09 [17], RRx‐001 [49], and Lonicerin [50], most have failed in clinical trials. To date, no NLRP3 inflammasome‐related inhibitors have been approved for clinical use by the FDA. The function requalification of FDA‐approved drugs provides a promising approach to rapidly develop potential inhibitors of inflammatory disease. Using this strategy, we found that CPX is a specific inhibitor of the NLRP3 inflammasome. CPX, a synthetic antifungal agent approved by the FDA, is also used in prescription shampoo and cream formulations [25, 51]. Currently, CPX, as an anticancer candidate drug, has been proven to be highly effective against gastric cancer [26] and hematologic malignancies [28]. CPX can also inhibit the production of cytokines (TNF‐α, IL‐1β, and IL‐6) in ischemic stroke rats [30]. Our study revealed that CPX significantly suppresses NLRP3 inflammasome‐driven caspase‐1 activation and IL‐1β secretion, indicating that CPX is an effective NLRP3 inflammasome inhibitor. Although our data show that the CPX's inhibitory effect on NLRP3 inflammasome activation is similar to that of MCC950, a strong NLRP3 inflammasome inhibitor, MCC950 has been discontinued in Phase II clinical trials due to its hepatotoxicity [52]. However, as a therapeutic drug approved by the FDA, CPX has high clinical safety and promising clinical applications. Indeed, IL‐1β is also produced by other activated inflammasomes or in an inflammasome‐independent manner, reflecting the diversity of host defenses [31, 32]. Nevertheless, CPX failed to inhibit the activation of the AIM2, NLRC4, pyrin, NLRP1, or NLRP6 inflammasomes, implying that CPX may not damage the host defense involved in these inflammasomes. Furthermore, our results demonstrate that CPX has remarkable therapeutic efficacy in LPS‐mediated sepsis and DSS‐induced colitis, both of which are driven by NLRP3 inflammasome activation [33, 34]. We also confirmed that CPX dramatically restrained NLRP3 inflammasome‐dependent IL‐1β secretion in human PBMCs. Notably, the beneficial effects of CPX on HFD‐induced metabolic disorders, an NLRP3 inflammation‐associated disease, were not observed in *NLRP3^−/−^* mice, indicating that the resolution of inflammation by CPX depends on the NLRP3 inflammasome. These results suggest that CPX has the potential to treat NLRP3 inflammasome‐related diseases.

The assembly signal is integral and critical for NLRP3 inflammasome activation and is controlled by numerous cellular events, including excessive mitochondrial ROS production [38], chloride ion efflux [36], and potassium efflux [37]. These upstream events have been validated as potential intervention targets for the NLRP3 inflammasome [36, 37, 38]. A previous study reported that ciclopirox, the active component precursor of CPX, attenuates NLRP3 inflammasome activation by abating mitochondrial ROS production [53]. However, our data demonstrate that CPX cannot inhibit mitochondrial ROS production, but also does not alleviate chloride ion and potassium efflux during NLRP3 inflammasome activation. This appearance might be caused by differences between the olamine salt and the precursor of ciclopirox. The complete assembled NLRP3 inflammasome is composed of the sensor NLRP3, adaptor ASC, and effector caspase‐1. ASC binds with NLRP3 through its PYD domain and with caspase‐1 via its CARD domain [1, 2, 4, 5]. The recruitment of ASC to NLRP3 is a significant step in NLRP3 inflammasome assembly, which promotes ASC oligomerization formation and subsequent caspase‐1 activation and IL‐1β secretion [1, 2, 16, 17]. In this study, we found that CPX hampers NLRP3‐ASC interaction, ASC oligomerization, and ASC speck formation. Interestingly, CPX also disrupted the NLRP3‐NEK7 interaction, another important step in NLRP3 inflammasome assembly [36]. Numerous studies have confirmed that NEK7 can directly bind to NLRP3 and is indispensable for NLRP3 inflammasome assembly and subsequent activation [21, 36, 54]. Previous studies have demonstrated that small‐molecule inhibitors suppress NLRP3 inflammasome activation by selectively disrupting NLRP3‐ASC interaction or NLRP3‐NEK7 interaction [21, 22, 55]. Our findings indicate that CPX directly hampers NLRP3 inflammasome assembly.

In this study, we focused on NLRP3 to further explore the mechanism by which CPX hampers NLRP3 inflammasome assembly. As a sensor and essential component of the NLRP3 inflammasome, NLRP3 oligomerization primarily determines its interactions with ASC and NEK7 [41, 49, 56]. Thus, NLRP3 is a common target for inhibiting NLRP3 inflammasome activation [13, 15, 16, 17, 18]. RRx‐001 binds to NLRP3 and subsequently prevents the interaction between NLRP3 and NEK7 to suppress NLRP3 inflammasome activation [49]. Costunolide targets NLRP3 to attenuate NLRP3 oligomerization and NLRP3 inflammasome assembly [57]. In this study, we found that CPX directly binds to NLRP3, rather than ASC or NEK7. Importantly, CPX also repressed NLRP3 oligomerization under NLRP3 inflammasome‐activating conditions. Therefore, we speculated that CPX‐mediated disruption of the NLRP3‐ASC and NLRP3‐NEK7 interactions depend on its ability to damage NLRP3 oligomerization by directly targeting NLRP3. Targeting IL‐1β is the available strategy for current clinical agent to treat NLRP3 inflammasome‐driven diseases; however, directly targeting NLRP3 with small‐molecule inhibitors exhibits preferable specificity and efficacy [16, 17, 18, 57]. CPX possesses the certain advantages. Notably, the NACHT domain appears to be the most likely target for small‐molecule inhibitors of the NLRP3 inflammasome [17, 18, 57]. Furthermore, our work showed that CPX binds to Y381 on the NACHT domain of NLRP3. A previous study identified the Y381 of NLRP3 is a direct target for inhibiting NLRP3 inflammasome activation [58]. Significantly, Y381 is located in the appropriate ATP‐binding pocket of NLRP3 [44]. The inhibitor docked into the ATP‐binding pocket of NLRP3 will blunt ATPase activity [44]. The ATPase activity of the NACHT domain is crucial for NLRP3 self‐association and oligomerization [18, 57]. Recent findings have confirmed that inhibitors can block NLRP3 inflammasome activation by suppressing ATPase activity [17, 18, 59]. Our study clearly demonstrated that CPX inhibits the ATPase activity of NLRP3 at doses of 5–20 µm. Thus, ATPase activity of NLRP3 may be the conclusive target of CPX to suppress NLRP3 inflammasome activation.

CPX is a therapeutic drug approved by the FDA with high clinical safety and excellent antifungal properties. Our results reveal previously unidentified properties of CPX as a specific and potent inhibitor of the NLRP3 inflammasome. We demonstrate that CPX targets the NACHT domain of NLRP3 to impair NLRP3 oligomerization and subsequent NLRP3 inflammasome assembly and full activation. These findings highlight the preventive and therapeutic effects of CPX against NLRP3‐induced inflammatory diseases, shedding valuable insights into the development of therapeutic agents for NLRP3 inflammasome‐driven diseases.

## 4 Experimental Section

### 4.1 Reagents and Antibodies

Ciclopirox olamine, imiquimod, TcdB toxin, and recombinant human NLRP3 were purchased from MedChemExpress (New Jersey, USA); Phorbol‐12‐myristate‐13‐acetate(PMA), LPS, nigericin, LDH activity assay kit, MitoTracker, MitoSOX, RIPA, human IgG, penicillin/streptomycin, NP40, and protein A+G agarose were supplied by Beyotime (Shanghai, China); MSU, SiO_2_, Alum, poly(dA:dT), pronase, disuccinimidyl suberate, and anti‐MYC were purchased from Sigma (St, Louis, USA); ATP, lipofectamine 2000, and *Salmonella typhimurium* (FLA‐ST) were supplied by InvivoGen (New Jersey, USA); and the ADP‐Glo Kinase assay kit was purchased from Promega (WI, USA). MCC950, Talabostat was purchased from Tsbiochem(Shanghai, China). Anti‐NLRP3, anti‐GPADH, horseradish peroxidase (HRP)‐linked horse anti‐mouse IgG, and HRP‐linked goat anti‐rabbit IgG were purchased from Proteintech (Wuhan, China); anti‐NEK7 and anti‐ASC were obtained from Santa Cruz Biotechnology (Santa Cruz, CA, USA); anti‐IL‐1β, anti‐NF‐κB, anti‐p‐NF‐κB, FITC conjugated anti‐mouse IgG, anti‐HA, and anti‐HIS were supplied by HuaBio (Hangzhou, China); anti‐GSDMD and anti‐IκB‐α were purchased from Cell Signaling Technology (Danvers, USA); anti‐caspase‐1 p20 was purchased from Adipogen (San Diego, CA, USA); and anti‐IL‐1β p17 and anti‐FLAG were purchased from Abmart (Shanghai, China).

### 4.2 Animals

Wild‐type (WT) and *NLRP3^−/−^* C57BL/6 mice were purchased from Cyagen (Suzhou, China). All mice were bred and housed under specific pathogen‐free conditions and a strict 12‐h light/12‐h dark cycle (lights on at 8:00 and off at 20:00) at 24 ± 2°C. All animal experiments were approved by the Animal Ethics Committee of Suining Central Hospital.

### 4.3 Cell Culture and Stimulation

HEK293T cells were grown in DMEM with 10% fetal bovine serum (FBS) containing 1% penicillin/streptomycin. THP‐1 cells were cultured in RPMI 1640 medium supplemented with 10% FBS and 1% penicillin/streptomycin. To generate human macrophages, THP‐1 cells were differentiated with 100 nm PMA and then incubated overnight. Primary BMDMs were harvested from C57BL/6 mice and cultured for 6 days in Dulbecco's modified Eagle's medium containing 10% phosphate buffered saline (PBS), 1% penicillin/streptomycin, and 20 ng/mL recombinant M‐CSF.

Human PBMCs were isolated from the fresh peripheral blood of 3 patients with acute gout and 3 healthy donors (all from Suining Central Hospital) using Lymphocyte Separation Medium. Written informed consent was obtained from all participants before inclusion in the study. The investigation was accomplished according to the guidelines of the Helsinki Declaration. Human PBMCs were cultured in RPMI 1640 medium supplemented with 10% FBS and 1% penicillin/streptomycin.

After priming with 500 ng/mL LPS for 3 h, the cells were treated with DMSO or inhibitors for 1 h and then stimulated with different inducers (10 µm nigericin for 1 h; 2.5 mm ATP for 40 min; 200 µg/mL MSU for 6 h; 300 µg/mL Alum for 6 h; 30 g/mL imiquimod for 3 h; 300 µg/mL SiO_2_ for 6 h) to activate the NLRP3 inflammasome. For AIM2, NLRC4, or Pyrin inflammasome activation, LPS‐primed THP‐1 cells were incubated with 0.25 µg/mL poly (dA:dT) for 4 h, 2.5 µg/mL flagellin from *Salmonella typhimurium* (FLA‐ST) for 4 h, or 0.5 µg/mL *Clostridium difficile* toxin B (TcdB) for 1 h to activate the other inflammasome. Human PBMCs were treated with DMSO or inhibitors for 1 h and then stimulated with Talabostat (10 or 30 µm).

### 4.4 Enzyme‐Linked Immunosorbent Assay (ELISA)

The levels of IL‐1β and TNF‐α in cell culture supernatants were measured using human ELISA kits (Multisciences, Hangzhou, China). IL‐1β and IL‐6 levels in mouse serum were quantified using a mouse ELISA Kit (Neobioscience, Shenzheng, China). All ELISAs were performed according to the manufacturer's instructions.

### 4.5 Western Blot and Co‐Immunoprecipitation (Co‐IP)

Total protein was extracted from cells and tissues using RIPA buffer. The protein concentrations of cell lysate were determined using a BCA protein assay kit (Beyotime Biotechnology), and equal amounts of protein from each sample were used. The cell culture supernatant was added to an equal volume of methanol and one‐quarter volume of chloroform. The mixture was vortexed for 1 min and centrifuged at 10 000 g for 10 min at 4°C. The supernatant was removed, and the pellet was resuspended in RIPA lysis buffer. For the Co‐IP assay, the cells were lysed in NP40 lysis buffer for 1 h at 4°C. The supernatants were gently shaken with 2 µg of the indicated primary antibody overnight, followed by incubation with protein A/G agarose at 4°C for 6 h. The samples were boiled in SDS sample buffer for 10 min at 100°C, separated by SDS‐PAGE, and transferred to a PVDF membrane. The membranes were blocked in blocking buffer for 10 min and then incubated overnight at 4°C with the primary antibodies. After three washes, the membranes were incubated with the conjugated secondary antibodies in blocking buffer for 1 h at room temperature. Subsequently, ECL western blotting chemiluminescent substrates were added, and the bands were acquired using an autoradiographic film.

### 4.6 Apoptotic Cell Death and LDH Release Assays

PI assays were conducted as previously described. Briefly, LPS‐primed cells in a 6‐well plate were washed with PBS and cultured in a basal salt solution containing 1 µg/mL PI and CPX for 1 h. After adding nigericin for 1 h, fluorescence microscopy was used to acquire cell images. The LDH release from cells was determined using the LDH Cytotoxicity Assay Kit (Beyotime Biotechnology) according to the manufacturer's instructions.

### 4.7 Analysis of Intracellular Ion Concentration

To measure the intracellular potassium (K^+^) and chloride (CL^−^) concentrations, THP‐1 cells were differentially treated with PMA in 10‐cm dishes. The overnight medium was replaced by DMEM and LPS for 3 h, and the cells were treated with CPX for 1 h. After stimulation with nigericin, the supernatants were carefully removed. THP‐1 cells were washed twice with PBS. The ion concentration was determined using assay kits according to the manufacturer's protocols.

### 4.8 Analysis of Mitochondrial ROS

The cells were incubated with prediluted MitoSOX (final concentration: 500 nm). The medium was removed and washed with PBS three times. The cells were fixed with 4% PFA in PBS for 15 min and then washed three times with PBST. Images were captured using laser‐scanning confocal microscopy.

### 4.9 Plasmid Construction and Transfection

NLRP3‐FLAG, NLRP3‐MYC, NLRP6‐FLAG, NEK7‐MYC, ASC‐HIS, pro‐caspase‐1‐HA, pro‐IL‐1β‐HA, NACHT‐FLAG, PYD‐FLAG, LRR‐FLAG, NACHT‐R167A‐FLAG, NACHT‐Y381A‐FLAG, NLRP3‐R167A‐FLAG, and NLRP3‐Y381A‐FLAG plasmids were purchased from RealGene Biotech (Shanghai, China). All constructs were tested and confirmed using DNA sequencing. Plasmids were transiently injected into HEK293T cells using Lipofectamine 3000 reagent according to the manufacturer's instructions.

### 4.10 ASC Speck Formation

THP‐1 cells were plated on chamber slides at 1 × 10^6^/mL and primed with LPS for 3 h the following day. After washing with PBS, the cells were treated with CPX for 30 min, followed by nigericin stimulation for 1 h. The cells were then washed three times with PBS buffer and fixed in 4% paraformaldehyde for 20 min and blocked with 1% BSA containing 0.3% Triton X‐100 for 1 h and subsequently incubated with the anti‐ASC at 4°C for 12 h. After that, cells were stained with the fluorescence‐labeled secondary antibody at room temperature for 60 min and sealed with DAPI. The fluorescence was imaged using confocal laser scanning microscopy.

### 4.11 ASC Oligomerization Assay

After nigericin stimulation, THP‐1 cells were lysed using NP40 for 30 min at 4°C. The precipitate was washed three times and resuspended in PBS following centrifugation. Resuspended pellets were incubated with 2 mm disuccinimidyl suberate for 30 min at room temperature. After centrifugation, the crosslinked pellets were resuspended in 30 µL of SDS loading buffer and analyzed by western blotting, as described previously.

### 4.12 Drug Affinity Responsive Target Stability (DARTS)

As previously described, THP‐1 or HEK293T cells were lysed using NP40 buffer, and cell lysates were mixed with CPX or dimethyl sulfoxide (DMSO) for 2 h at room temperature. After binding, each lysate mixture containing equal amounts of proteins was digested by pronase at room temperature for 10 min, and the process was halted with sodium dodecyl sulfate loading buffer for boiling and immunoblotting.

### 4.13 Cellular Thermal Shift Assay (CETSA)

THP‐1 cells were collected and lysed in NP40 buffer containing protease inhibitor cocktail and centrifuged at 12 000 g for 15 min at 4°C. The protein lysate was aliquoted and incubated with CPX or DMSO for 2 h at room temperature. Subsequently, the treated cell lysates were equally divided into PCR tubes and heated separately at different temperatures (50°C, 55°C, 60°C, 65°C, 70°C, and 75°C) for 6 min, followed by cooling on ice for 5 min. The soluble fractions were separated after centrifugation for 25 min (20 000 × g, 4°C) and collected to detect the target protein by western blotting.

### 4.14 Surface Plasmon Resonance (SPR) Experiment

The binding affinity of CPX for the human NACHT domain of NLRP3 was determined using a Biacore T200 instrument (GE Healthcare). The activator was prepared by mixing 0.4 m EDC and 0.1 m NHS immediately before injection. Recombinant proteins were immobilized on the sensors and blocked with 1 m ethanolamine hydrochloride. A concentration gradient of CPX (1.56, 3.12, 6.25, 12.5, 25, and 50 µm) was prepared in running buffer (PBS containing Tween 20% and 5% DMSO), with the same analyte buffer used for the six concentrations. Six concentrations of CPX were injected at a flow rate of 30 µL/min for a 120‐s dissociation phase, followed by a 200‐s dissociation phase. The corresponding response values were recorded by the instrument, and the affinity constant (KD) value was calculated using Biacore analysis software.

### 4.15 Molecular Docking and Molecular Dynamics Simulation (MDS)

The NLRP3 receptor protein (PDB ID: 7ALV) was acquired from the Protein Data Bank database. The three‐dimensional (3D) structure of CPX was sourced from the PubChem database. The molecular docking of CPX and NLRP3 was performed using Autodock4 software, with the following docking parameters: NLRP3: Center (X, Y, and Z) = (16.283, 33.837, and 131.569); size (X x Y x Z) = (88, 90, and 116), and the docking method set as semi‐flexible. This configuration results in a 3D grid used to obtain the search space for MDS, and the optimal docking pose was selected according to the docked free energy. Following visualization in PyMOL 4.6.0, the optimal docked structure (lowest free energy) was used for MDS with Gromacs2024.4 software using Amber14sb force field. The MDS was run for 100 ns, and the root mean square deviation (RMSD), radius of gyration (Rg), root mean square fluctuation (RMSF), and hydrogen bond curves were calculated using Gromacs2024.4.

### 4.16 NLRP3 Oligomerization

NLRP3 oligomerization was evaluated using semi‐denaturing detergent agarose gel electrophoresis (SDD‐AGE) and Native‐PAGE, as described previously. For SDD‐AGE analysis, cells were suspended in Triton X‐100 lysis buffer (0.5% Triton X‐100, 50 mm Tris‐HCl, 10% glycerol, 150 mm NaCl, 1 mm PMSF, and protease inhibitor cocktail). After centrifugation, the lysate was collected and resuspended in sample buffer (0.5X TBE, 2% SDS, and 10% glycerol containing 0.0025% bromophenol) and electrophoresed on a vertical 1.5% agarose gel in running buffer (1X TBE and 0.1% SDS) for 1 h at 4°C at a constant voltage of 100 V. The proteins were then transferred to the PVDF membrane for immunoblotting. For native PAGE analysis, the precipitate was collected and incubated with RIPA buffer. After complete dissolution of the precipitate, 6X native‐PAGE loading buffer was added.

### 4.17 NLRP3 ATPase Activity Assay

Purified recombinant human NLRP3 protein was incubated with different concentrations (5, 10, 20 µm) of CPX in the reaction buffer for 1 h at 37°C. Then, the ADP‐Glo Kinase Assay Kit (Promega) was employed to measure the quantification of ATP according to the manufacturer's protocol, and the luminescent intensity was analyzed.

### 4.18 LPS‐Induced Sepsis

Male C57BL/6 mice aged 10 weeks were injected intraperitoneally with vehicle, 10, or 20 mg/kg CPX 1 h before injection of 20 mg/kg LPS to induce an endotoxemia model. For liver function markers (ALT and AST) and cytokine (IL‐1β and IL‐6) assay, serum samples were collected at 6 h and centrifuged at 3500 rpm for 10 min. Liver samples were collected for H&E staining and western blot analysis. The survival of mice was monitored for 80 h after LPS injection.

### 4.19 DSS‐Induced Colitis

10‐week‐old male C57BL/6 mice were treated with 3% DSS in their drinking water for 6 days and then provided with normal drinking water for 4 days. The control mice were given regular drinking water. Mice received a daily intraperitoneal injection of vehicle or 20 mg/kg CPX for 10 days. The body weight, stool, and body posture of mice were monitored daily to assess the disease activity index (DAI) during the experiment. The DAI was scored on a scale of 0 to 4, as previously described. Mice were euthanized at the indicated time points, and colons were harvested for length measurement, histological examination, cytokine quantification, and western blot analysis.

### 4.20 HFD‐Induced Metabolic Disorder

Wild‐type and *NLRP3^−/−^* male C57BL/6 mice (6 weeks old) with similar body weights were randomly assigned to different groups. To establish the HFD‐induced diabetic model, wild‐type and *NLRP3^−/−^* mice were maintained on a 60 kcal% fat diet for 12 weeks (HFD, Xietong Shengwu Ltd., China) throughout the experiment. Mice received a daily intraperitoneal injection of either vehicle or CPX (20 mg/kg) in the last 4 weeks.

### 4.21 Glucose Tolerance or Insulin Tolerance Test

For the glucose tolerance test (GTT), mice were fasted for 14 h and then intraperitoneally administered glucose at 1.5 g/kg. Blood samples were collected from the tail vein at different time points, and blood glucose levels were measured using a Nova Biomedical StatStrip Xpress glucometer. For the insulin tolerance test (ITT), mice were fasted for 4 h and then intraperitoneally administered human recombinant insulin (Novo Nordisk) (1 IU/kg). Blood glucose levels were measured from the tail vein at different time points.

### 4.22 Hematoxylin‐Eosin (H&E) Staining

Fresh mouse liver and colon tissue samples were fixed in 4% paraformaldehyde for stabilization, followed by embedding in paraffin, freezing sectioning, and H&E staining.

### 4.23 Statistical Analysis

All data are presented as mean ± standard deviation (SD). The unpaired Student's *t*‐test or two‐way analysis of variance (ANOVA) was used to perform statistical calculations between groups using GraphPad Prism 9.5 (GraphPad, San Diego, CA, USA). The survival rates of mice were expressed as Kaplan–Meier survival curves, and the difference between survival rates was analyzed using the log‐rank (Mantel–Cox) test. P‐values < 0.05 were considered statistically significant.

## Author Contributions

Y.Z. designed the experiments, reviewed data, and supervised the research and overall study. X.X., K.Z., and H.Y. collected data and analyzed the data in this study. Y.Z., X.X., and H. Y. wrote the manuscript. X.X. and K.Z. performed in vivo experiments. H.J., L.L., and H.L. performed in vitro experiments. All authors discussed the results and participated in manuscript preparation and editing.

## Funding

This study was supported by the National Natural Science Foundation of Sichuan Province (Grant No. 2024NSFSC1766). Sichuan Medical Association Research Project (Grant No. Q23026; Q20250082). Sichuan Provincial Administration of Traditional Chinese Medicine Research Project (Grant No. 2024MS639). Suining Health Science and Technology Program (Grant No. 24CJDFB03; 24CJDFB14; 25ZDJB10; 25CJDFB01).

## Conflicts of Interest

The authors declare no conflicts of interest.

## Supporting information




**Supporting File**: advs75704‐sup‐0001‐SuppMat.doc.

## Data Availability

Data sharing not applicable to this article as no datasets were generated or analysed during the current study.
