# Supplementary material for: Ciclopirox Olamine Inhibits the NLRP3 Inflammasome to Alleviate Inflammatory Diseases
Source: Adv Sci (Weinh). 2026 May 19;13(40):e75704. doi: 10.1002/advs.75704 (PMC13336122; doi:10.1002/advs.75704)
Supplement: Supplementary file 1 — Supporting File: advs75704‐sup‐0001‐SuppMat.doc. [file ADVS-13-e75704-s001.doc]

**Ciclopirox olamine inhibits the NLRP3 inflammasome to alleviate inflammatory diseases**

Xinyu Xia^1^* Hui You^2^* Ke Zhang^1^* Hui Jiang^3^ Hongping Liu^1^ Lianghua Liu^3^ Aijie Zhang^4✉^ You Zhou^1✉^

1 Department of Medical Laboratory, Suining Central Hospital, Suining, 629000, China.

2 Department of Ophthalmology, Suining Central Hospital, Suining, 629000, China.

3 The biology bank of Suining Central Hospital, Suining Central Hospital, Suining, 629000, China.

4 Basic Laboratory, Suining Central Hospital, Suining, 629000, China.

✉Corresponding author: Aijie Zhang [(aijie8666@163.com) (ORCID: 0000-0002-0241-1845);](mailto:(aijie8666@163.com) (ORCID: 0000-0002-0241-1845);) You Zhou ([zhouy20@lzu.edu.cn](mailto:zhouy20@lzu.edu.cn))

*Xinyu Xia, Hui You, Ke Zhang contributed equally to this paper

THP-1 cells were seeded in 96-well plates and primed with for 3 hours, and then treated with 190 compounds at a concentration of 10 µM, followed by stimulation with 10 µM nigericin for 1 hour. The supernatant was collected, and IL-1β levels were measured using human ELISA kits.

The inhibition rate was calculated using the formula:

Inhibition Rate (%)
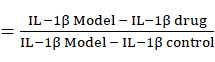
 * 100%

**
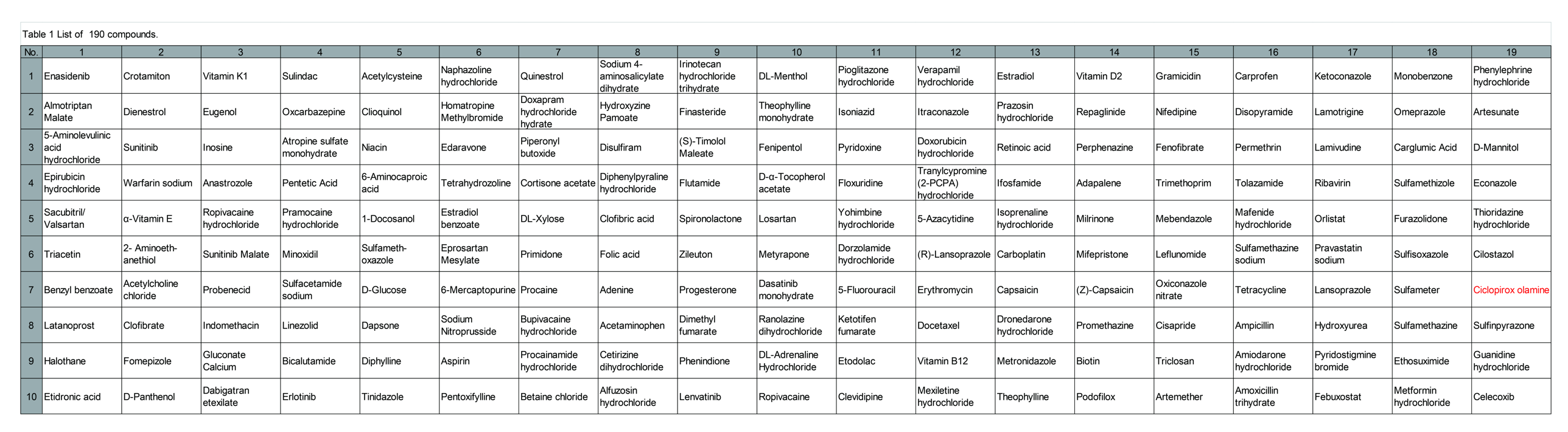
**

**Supplementary Figure. 1** List of 190 FDA-approved drugs.

**
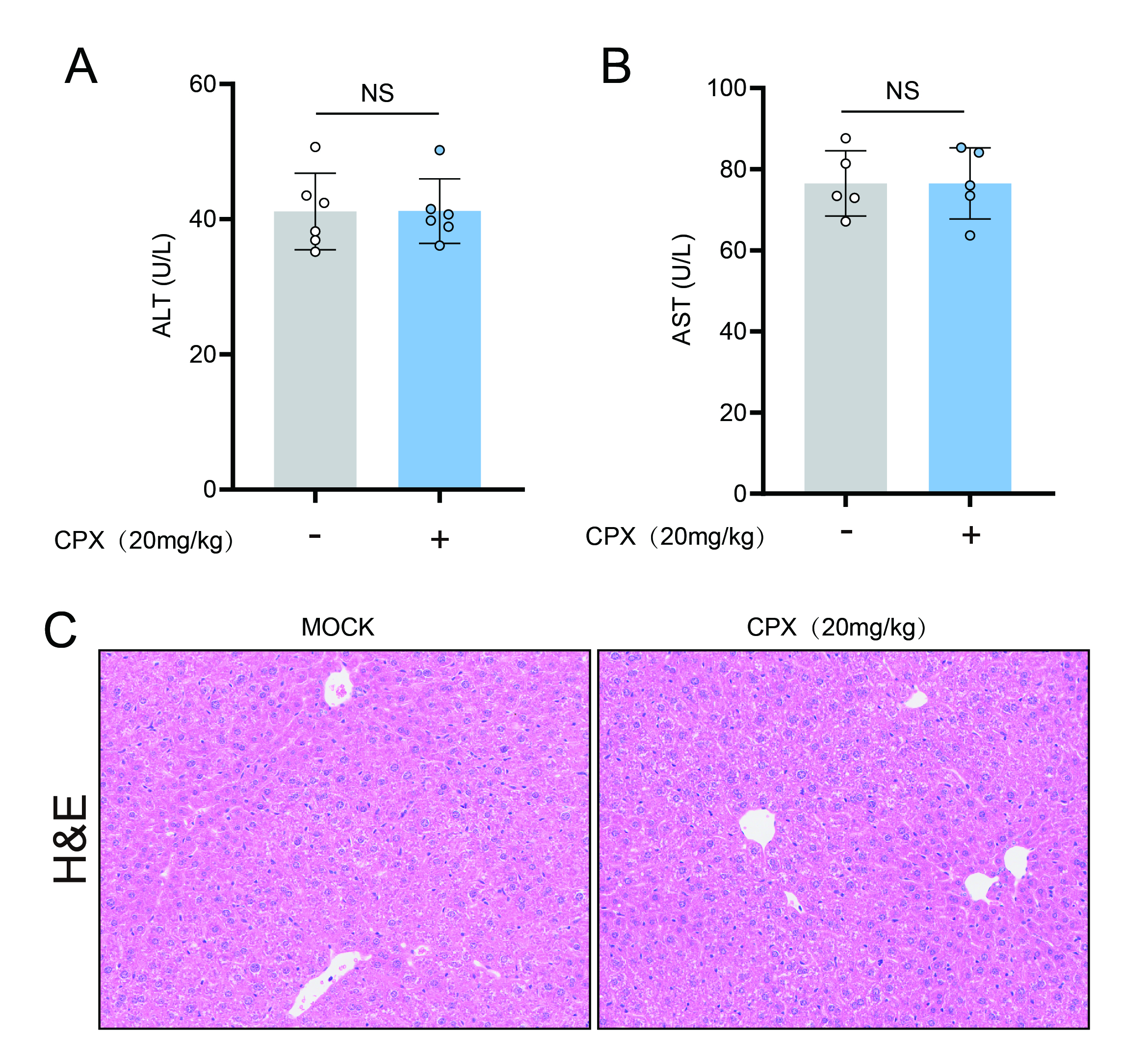
**

**Supplementary Figure. 2** (A-C) C57BL/6 mice received a daily intraperitoneal injection of either vehicle or CPX (20 mg/kg) in 3 days (n = 6). The activity of plasma ALT (A) and AST (B), and representative H&E staining of livers (C) of mice.

**
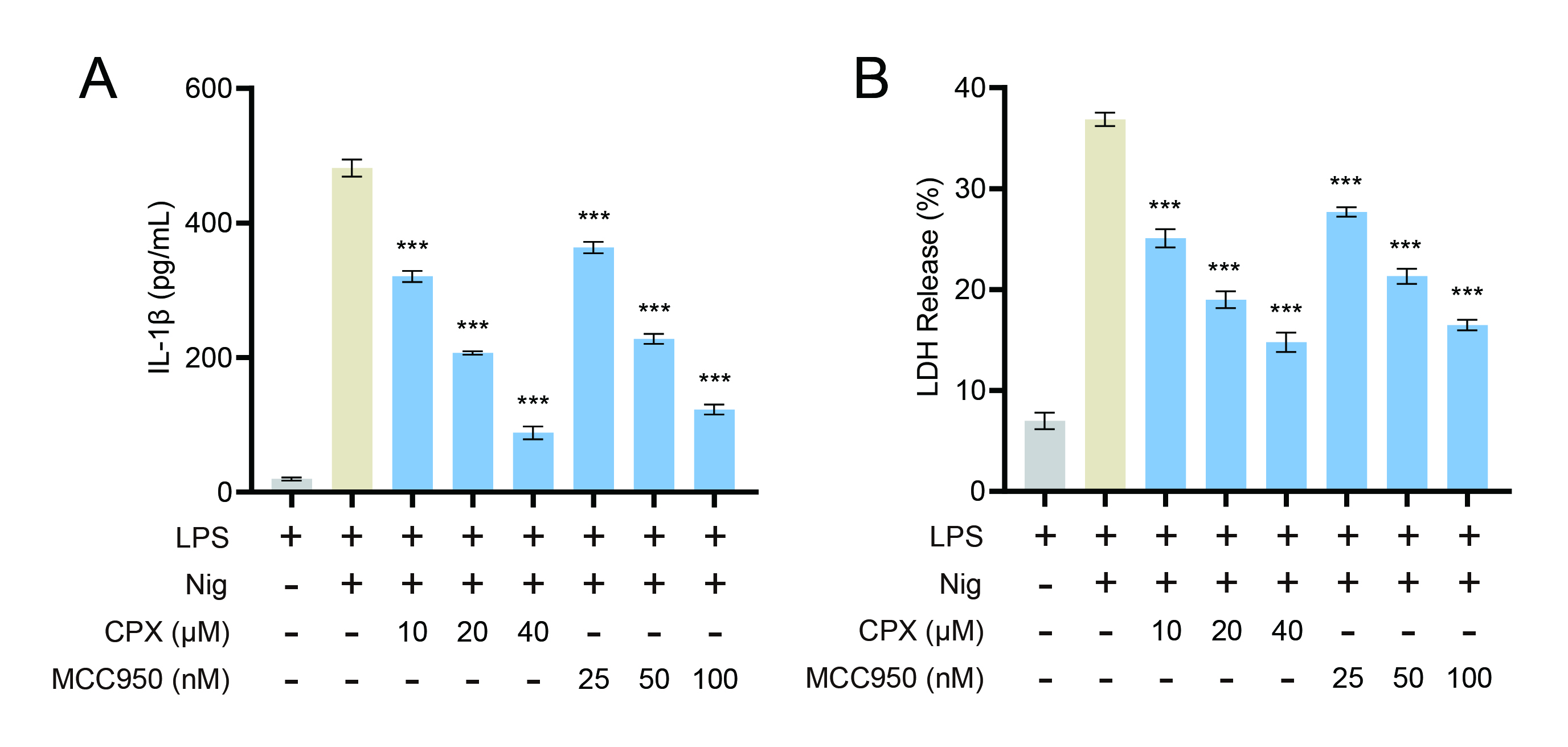
**

**Supplementary Figure. 3** THP-1 cells were first primed with LPS (3 h) and then treated with or without CPX(10, 20, 40μM) or MCC950(25, 50, 100nM)for 1 h, followed by stimulation with nigericin for 1 h. ELISA of IL-1β in Sup(A), and release of LDH from cells(B). Date from three independent experiments(Values are mean±SD). Statistical analyses were carried out via one-way ANOVA, ***p < 0.001.

**
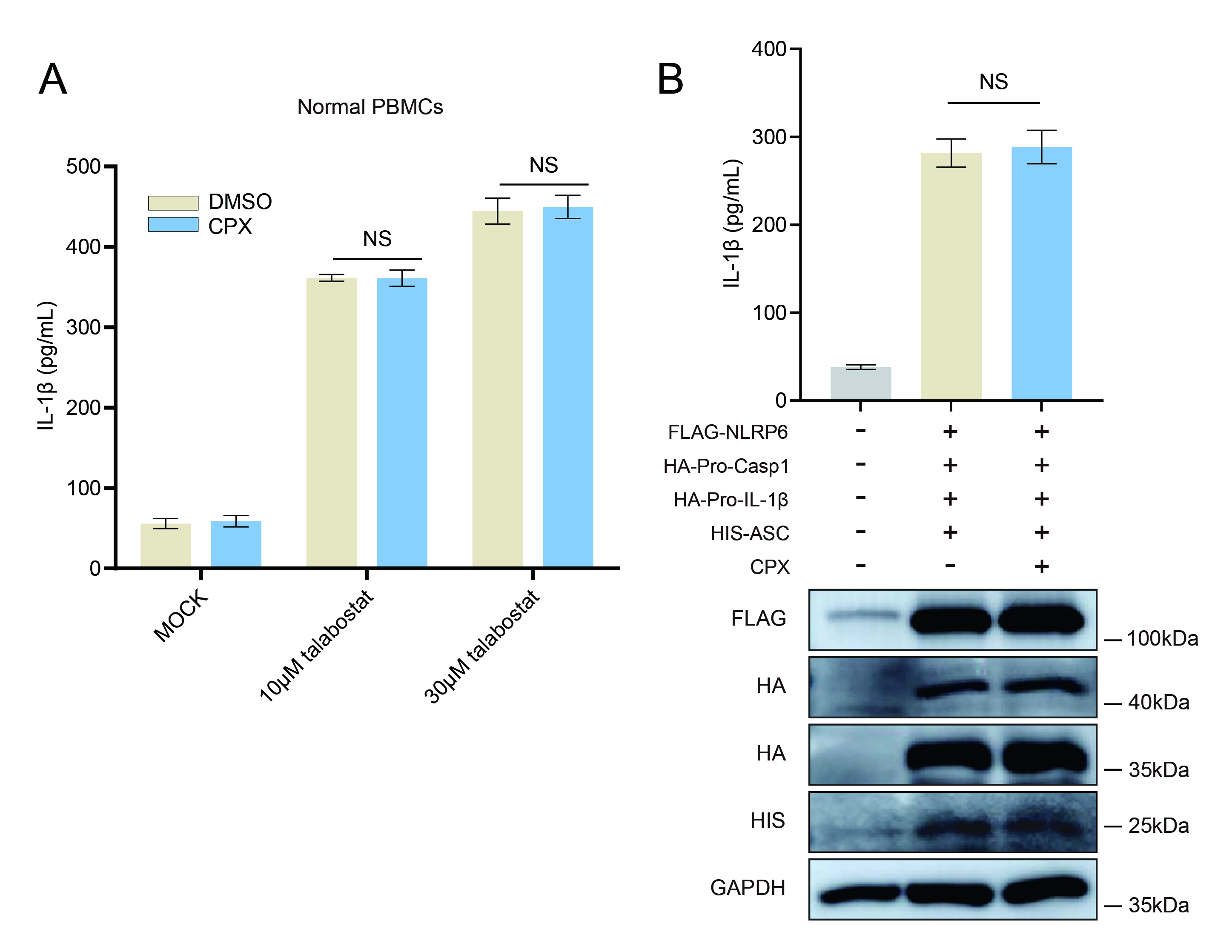
**

**Supplementary Figure. 4** (A) PBMCs were treated with 20 μM CPX for 1 h, and then stimulated with Talabostat(10 μM or 20 μM). ELISA of IL-1β in Sup. (B) ELISA of IL-1β in Sup from HEK293T cells transfected with HIS-ASC, FLAG-NLRP6, HA-IL-1β, HA-pro-caspase-1plasmids and treated with 20 μM CPX. Date from three independent experiments(Values are mean±SD). Statistical analyses were carried out via two-way ANOVA, NS not significant.

**
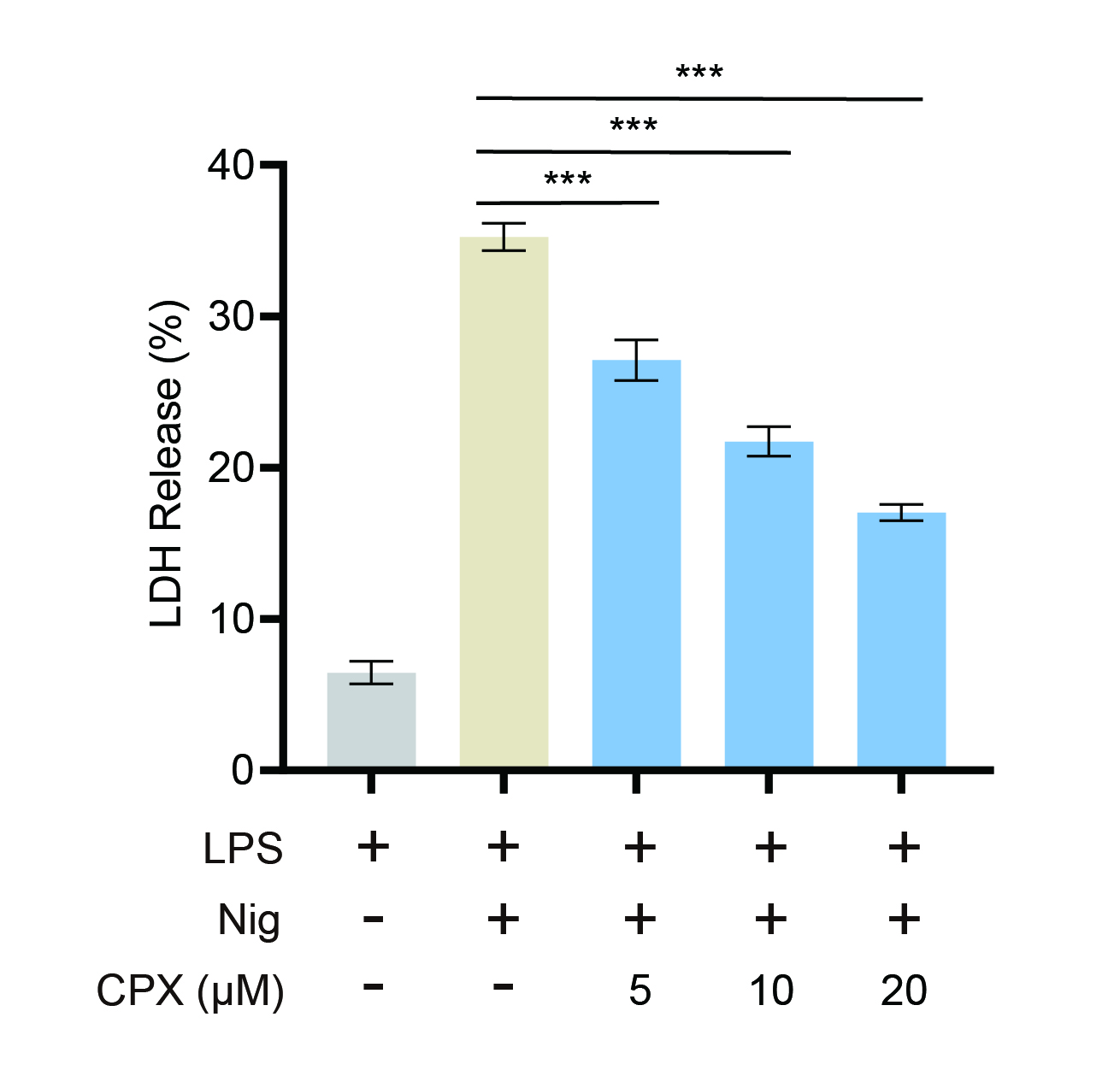
**

**Supplementary Figure. 5** The release of LDH from LPS-primed THP-1 cells, pre-treated with various doses of CPX or DMSO and then stimulated with nigericin.

**
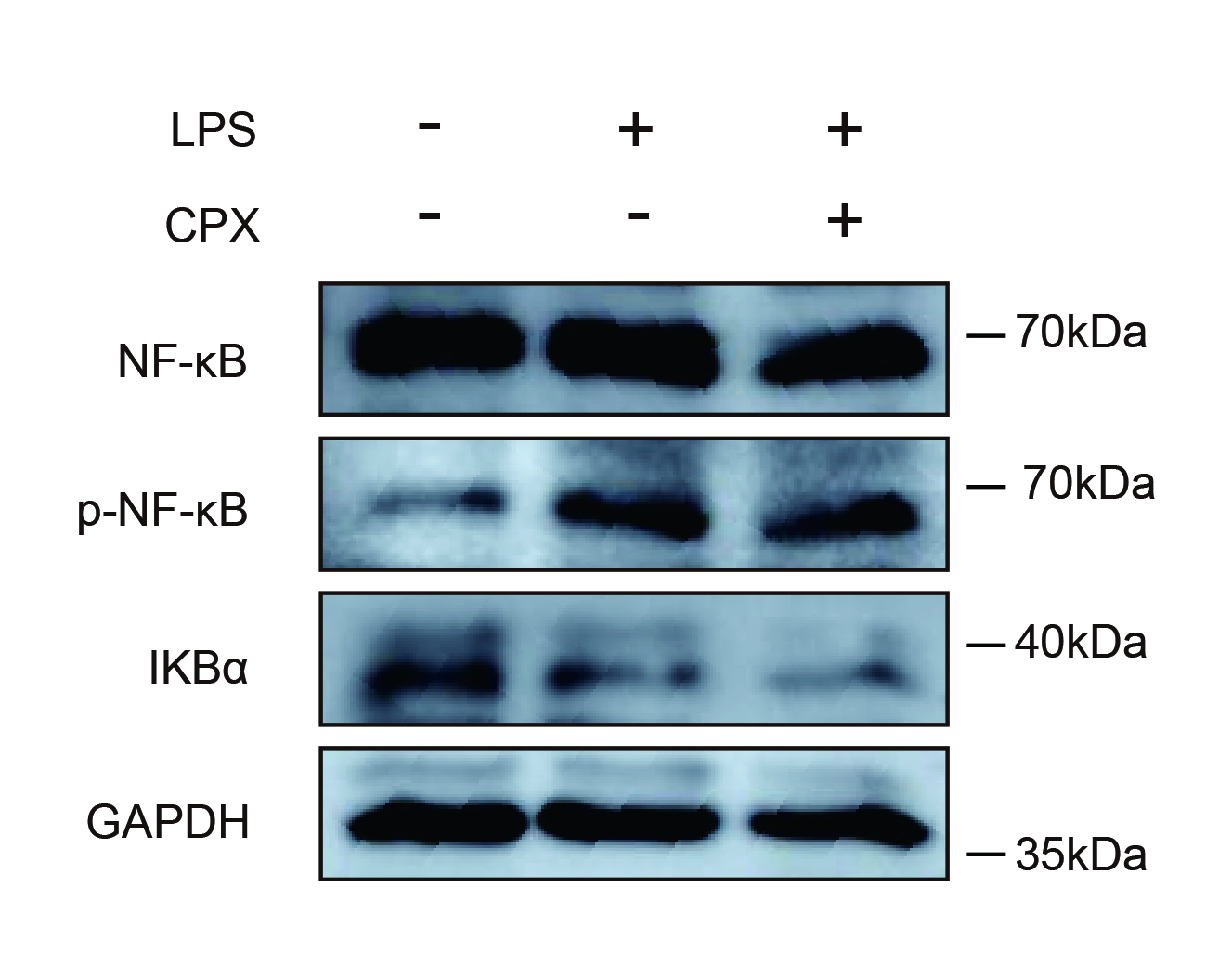
**

**Supplementary Fig. 6** THP-1 cells were first primed with LPS (30min) and then treated with or without 20 μM CPX for 1 h. Immunoblot analysis of NF-κB，p-NF-κB, and IKBα in Lys.

**
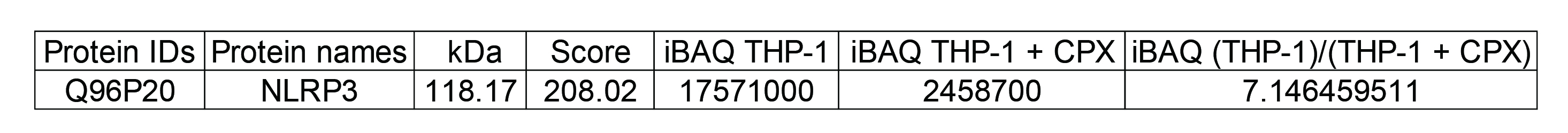
**

**Supplementary Figure. 7** NLRP3 was identified as the target protein of CPX by mass spectrogram.

**
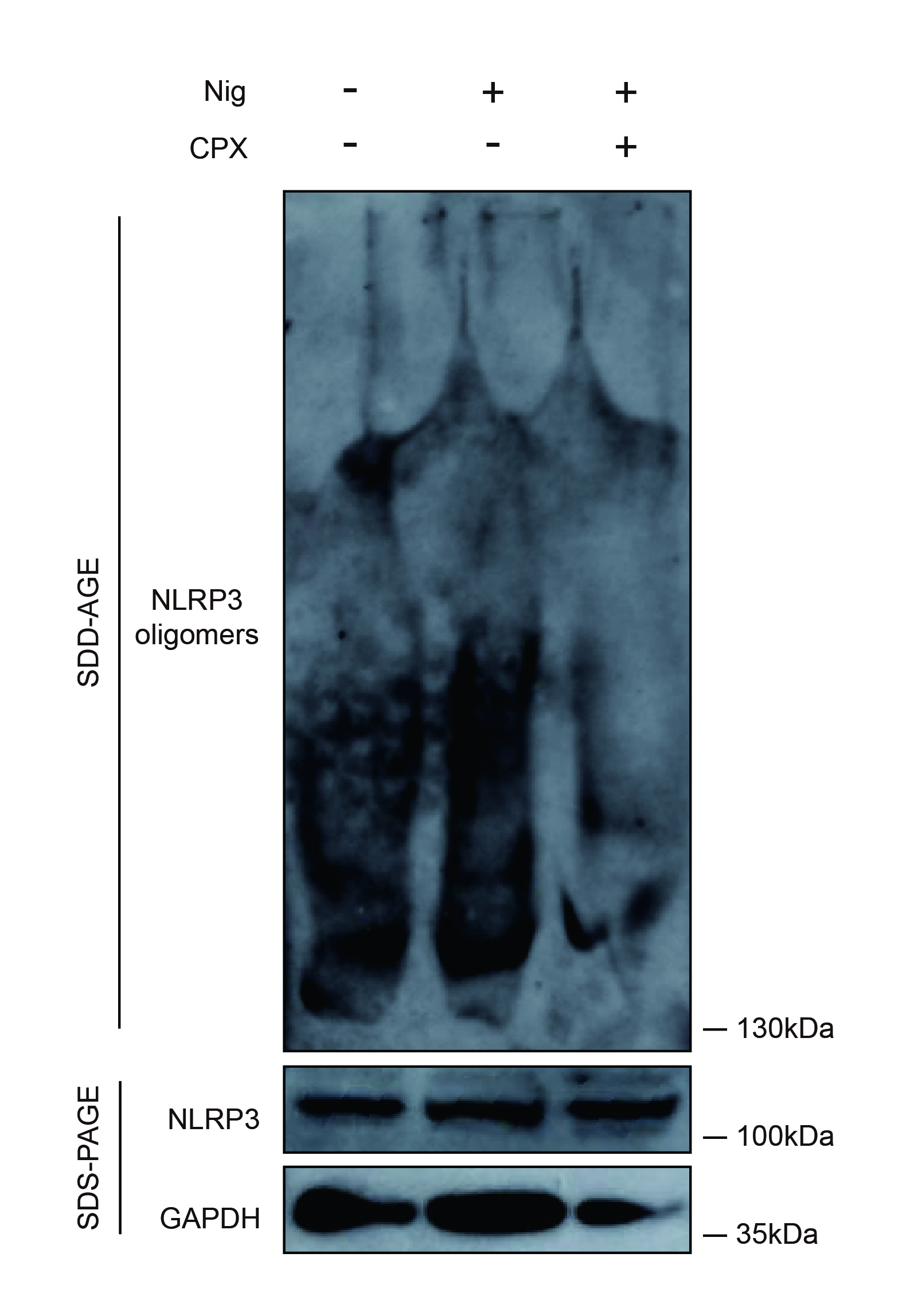
**

**Supplementary Figure. 8** The oligomerization of NLRP3 in THP-1 cells treated with CPX analyzed by SDD-AGE.

**
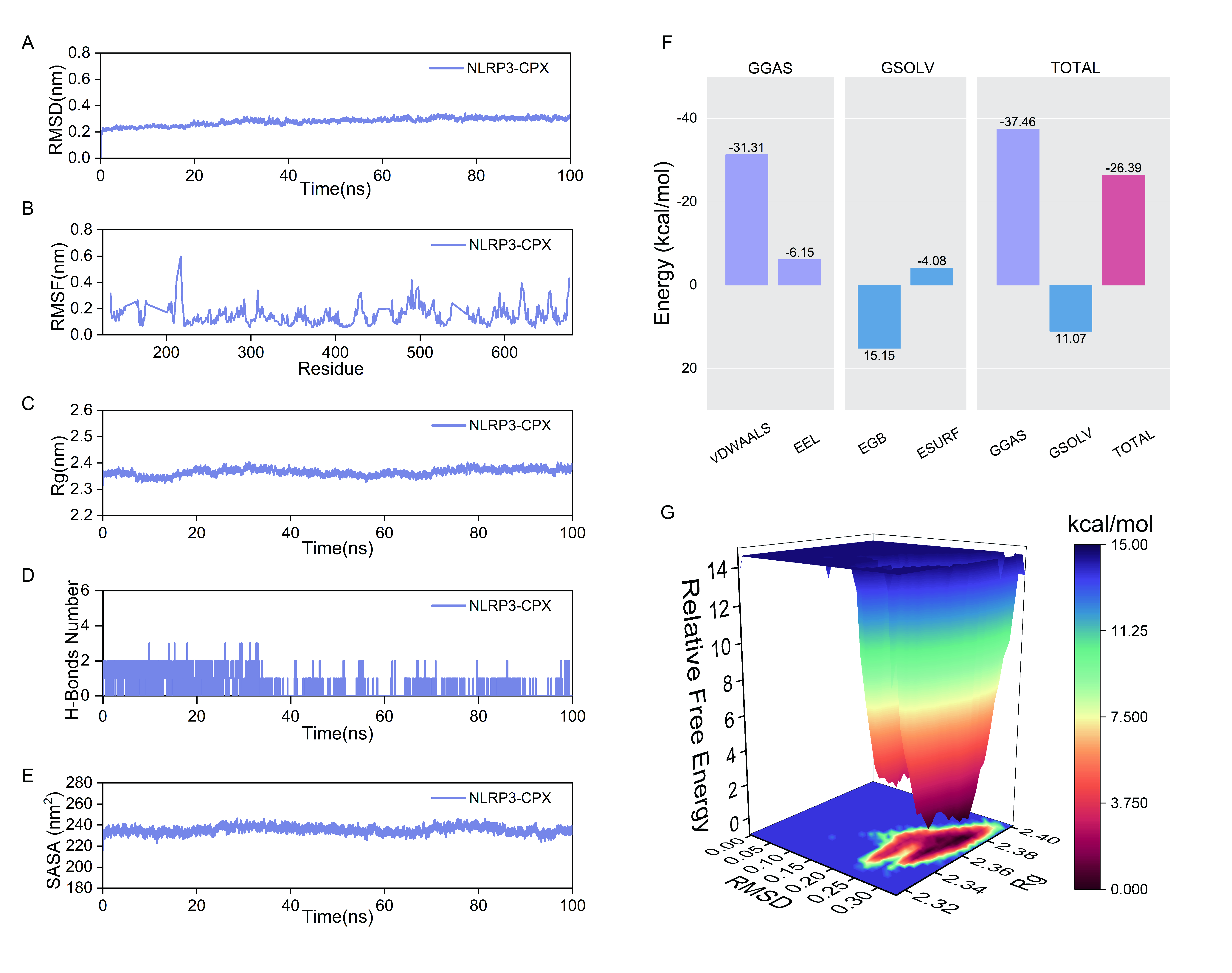
**

**Supplementary Figure. 9** Molecular dynamics simulation. (A) RMSD curve, (B) RMSF curve, (C) Rg curve, (D) H-bonds plot, (E) SASA plot, (F) Plots of MM/GBSA binding energy, (G) Plots of Gibbs FEL.

**
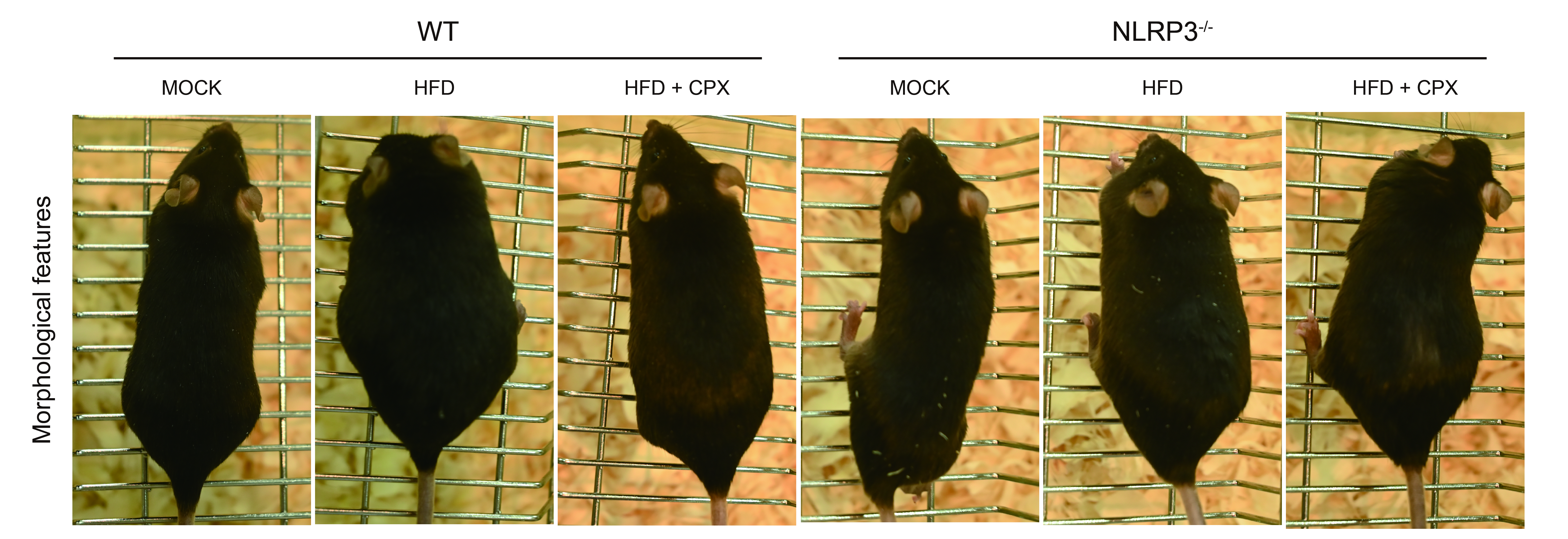
**

**Supplementary Figure. 10** Wild-type and *NLRP3^-/-^* C57BL/6 were maintained on an HFD for 12 weeks throughout the experiment. Mice received a daily intraperitoneal injection of either vehicle or CPX (2 mg/kg) in the last 4 weeks(*n*=6). Representative morphological features from each group.
